# 7D High‐Dynamic Spin‐Multiplexing

**DOI:** 10.1002/advs.202402378

**Published:** 2024-06-28

**Authors:** Yue Qin, Hao Guo, Sebastian Pazos, Mengzhen Xu, Xiaobing Yan, Jianzhong Qiao, Jia Wang, Peng Zhou, Yang Chai, Weida Hu, Zhengqiang Zhu, Zhonghao Li, Huanfei Wen, Zongmin Ma, Xin Li, Mario Lanza, Jun Tang, He Tian, Jun Liu

**Affiliations:** ^1^ State Key Laboratory of Dynamic Measurement Technology Shanxi Province Key Laboratory of Quantum Sensing and Precision Measurement North University of China Taiyuan 030051 P. R. China; ^2^ Materials Science and Engineering Program Physical Science and Engineering Division King Abdullah University of Science and Technology (KAUST) Thuwal 23955‐6900 Saudi Arabia; ^3^ State Key Laboratory of Hydroscience and Engineering Tsinghua University Beijing 100084 China; ^4^ National‐Local Joint Engineering Laboratory of New Energy Photovoltaic Devices Key Laboratory of Brain‐Like Neuromorphic Devices and Systems of Hebei Province College of Electron and Information Engineering Hebei University Baoding 071002 China; ^5^ School of Automation Science and Electrical Engineering Beihang University Beijing 100191 China; ^6^ State Key Laboratory of ASIC and System School of Microelectronics Fudan University Shanghai 200433 China; ^7^ Department of Applied Physics The Hong Kong Polytechnic University Hong Kong 999077 China; ^8^ State Key Laboratory for Infrared Physics Shanghai Institute of Technical Physics Chinese Academy of Sciences Shanghai 200083 China; ^9^ School of Integrated Circuits and Beijing National Research Center for Information Science and Technology (BNRist) Tsinghua University Beijing 100084 China; ^10^ Beijing Institute of Aerospace Control Devices Beijing 100094 China

**Keywords:** data storage, encryption, laser direct writing, multiplexing, silicon carbide color centers

## Abstract

Multiplexing technology creates several orthogonal data channels and dimensions for high‐density information encoding and is irreplaceable in large‐capacity information storage, and communication, etc. The multiplexing dimensions are constructed by light attributes and spatial dimensions. However, limited by the degree of freedom of interaction between light and material structure parameters, the multiplexing dimension exploitation method is still confused. Herein, a 7D Spin‐multiplexing technique is proposed. Spin structures with four independent attributes (color center type, spin axis, spatial distribution, and dipole direction) are constructed as coding basic units. Based on the four independent spin physical effects, the corresponding photoluminescence wavelength, magnetic field, microwave, and polarization are created into four orthogonal multiplexing dimensions. Combined with the 3D of space, a 7D multiplexing method is established, which possesses the highest dimension number compared with 6 dimensions in the previous study. The basic spin unit is prepared by a self‐developed laser‐induced manufacturing process. The free state information of spin is read out by four physical quantities. Based on the multiple dimensions, the information is highly dynamically multiplexed to enhance information storage efficiency. Moreover, the high‐dynamic in situ image encryption/marking is demonstrated. It implies a new paradigm for ultra‐high‐capacity storage and real‐time encryption.

## Graphical abstract

A 7D Spin‐multiplexing technique is proposed based on four independent attributes and four independent physical effects of spin structures. The highest number of dimensions is achieved, and the information is highly dynamically multiplexed to enhance information storage efficiency. High‐dynamic in situ image encryption/marking is demonstrated. It implies a new paradigm for ultra‐high‐capacity storage and real‐time encryption.

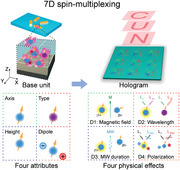

## 1 Introduction

Multiplexing technology provides multiple orthogonal data dimensions and channels for information storage and application, significantly expanding the physical dimension/freedom of information application.^[^ ^1^, ^2^, ^3^, ^4^, ^5^, ^6^, ^7^, ^8^, ^9^, ^10^ ^]^ It has broad prospects in high‐capacity information storage and encryption,^[^ ^1^, ^2^, ^7^ ^]^ optical holography,^[^ ^3^, ^5^, ^7^ ^]^ mechanical control, neural behavior simulation, etc.

Currently, multiplexing technology encompasses optical multiplexing,^[^ ^1^, ^2^, ^3^, ^5^, ^7^, ^8^, ^9^ ^]^ electrical multiplexing,^[^ ^4^ ^]^ and network algorithm simulation. As the mainstream multiplexing platform, metasurface utilizes the spatial variation of phase discontinuities caused by a single‐layer subwavelength antenna array.^[^ ^1^, ^2^, ^3^, ^4^, ^5^ ^]^ By utilizing rich light attributes such as wavelength, polarization, and orbital angular momentum (OAM), optical multiplexing provides numerous degrees of freedom for high‐capacity information storage, image encryption, and optical holography. Optical multiplexing has extensively explored various physical dimensions of light, yet it has nearly exhausted all available options, and the current maximum reported number of dimensions is six, (including three dimensions of space).^[^ ^2^, ^3^, ^7^ ^]^ The development of new multiplexing dimensions is challenging due to the limitations imposed by the metasurface structure parameters and the limited degree of freedom of light.^[^ ^10^, ^11^ ^]^ The limitations of electrical multiplexing technology are determined by factors such as the scattering angle, frequency, and phase of the electromagnetic field.^[^ ^10^, ^11^ ^]^ Currently, there exists only one additional dimension for electrical multiplexing.^[^ ^4^ ^]^ Although significant progress has been made in the dimension of existing multiplexing technology, the number of dimensions that can be practically applied is limited by the degree of freedom of interaction between light and material structure parameters.^[^ ^4^, ^11^ ^]^ The exhaustion of available dimensions has become a major constraint on the development of multiplexing.^[^ ^3^ ^]^ The exploration of multiplexing technology based on the new mechanism is therefore crucial for the development of next‐generation information storage, encryption, and other applications.

Here, we have constructed four new additional dimensions of multiplexing through the utilization of the Zeeman effect,^[^ ^12^, ^13^, ^14^, ^15^, ^16^ ^]^ photoluminescence (PL) effect,^[^ ^12^, ^13^, ^17^, ^18^, ^19^, ^20^, ^21^, ^22^ ^]^ Rabi oscillation effect,^[^ ^12^, ^17^, ^23^, ^24^, ^25^ ^]^ and dipole transition effect^[^ ^18^, ^19^, ^26^, ^27^, ^28^, ^29^ ^]^ of spin structures. The design of four orthogonal multiplexing dimensions and corresponding channels is achieved by introducing four physical structural parameters, namely axial direction, type, height, and dipole direction. These codes of the above parameters are read by controlling four physical quantities including magnetic field intensity, wavelength, microwave pulse duration time, and polarization respectively. This approach enables high‐capacity encoding. We prepared spin structures (divacancy color center) with rich physical structural parameters in laser‐induced silicon carbide (SiC) nanocrystals on Polydimethylsiloxane (PDMS)^[^ ^6^, ^20^, ^30^, ^31^, ^32^, ^33^, ^34^, ^35^, ^36^, ^37^ ^]^ to realize the 7D multiplexing by combining it with the three dimensions of space. The high transparency and flexibility of spin‐multiplexing encoding device (SMED) enables it to be seamlessly integrated with vision systems, thereby achieving real‐time in situ encryption/watermarking and immediate transmission of captured images. Therefore, the special features of SMED, including its rapid encoding capacity as well as encryption capabilities, have been validated. Moreover, easy integration, low cost, simple processing and mature fully electronic control scheme of SMED enable it to emerge as the fastest multiplexing technology to achieve engineering applications.

## 2 Mechanism of Spin‐Multiplexing

**Figure 1:**
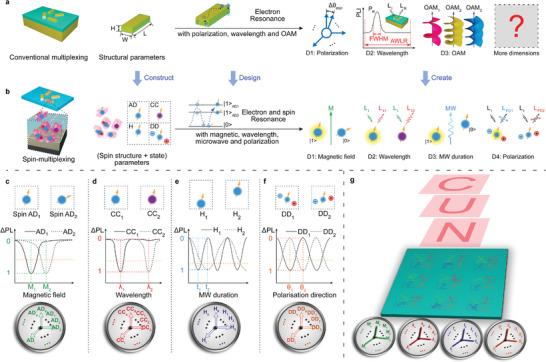
Overview of conventional multiplexing and 7D Spin‐multiplexing. a) Conventional multiplexing. b) Our proposed 7D Spin‐multiplexing. Among them, the blue polygons represent the SiC crystals. The balls and arrows represent the electrons and spin properties of spin structures, respectively. The direction of arrow, color of ball, vertical position, and direction of surrounding balls represent the axial direction (AD), color center type (CC), height (H), and dipole direction (DD) of spin structures, respectively. |1>_AD1_, |1>_AD2_ and |0> are three energy levels of spin structures. Yellow circular light indicates the code of corresponding spin structure is 1 (the electron transitions to the spin level |1>), and the rest of the spin structures are coded 0. c–f) magnetic field (c), wavelength (d), microwave (e) and polarization dimension (f) of 7D Spin‐multiplexing. The upper, middle, and lower parts of each graph are respectively the spin structures of different properties, the spectrum of the spin structure, and the concrete representation of the spin properties (dashboard). g) Externally controlled physical quantities, whole structure, and PL image with each dimension of 7D Spin‐multiplexing.

As illustrated in **Figure** **1**a,b, created from the conventional multiplexing technology, the spin‐multiplexing encoding basic unit is composed of spin structures and spin state parameters, which were four kinds of encoding parameters (color center type, spin axis, position distribution, and dipole direction) rather than structure parameters (confined to three dimensions). Meanwhile, the encoding mechanism of spin resonance was expanded from the conventional electron resonance to establish the new dimension. The encoding mode of four additional dimensions is established based on the physical effects of electron/spin structure. The coding unit can be independently encoded by these four physical quantities by modeling a one‐to‐one correspondence between these structure/state parameters and four physical quantities. The encoding dimension is expanded to seven dimensions containing three dimensions of space. At the same time, because the parameters of intensity/polarization degree/band of these four physical quantities can be controlled by an electrical signal, information can be dynamically multiplexed.

The multiplexing coding scheme based on four physical quantities is shown in Figure 1c–f. First, four orthogonal multiplexing dimensions and channels are constructed based on four kinds of parameters: axial direction (Figure 1c), type (Figure 1d), height (Figure 1e), and dipole direction (Figure 1f) of spin structures. Subsequently, the above four dimensions are associated with the four physical quantities of the magnetic field, wavelength, microwave pulse duration time, and light polarization respectively through four physical effects (spin magnetic resonance, PL, Rabi oscillation, and dipole transition). Finally, code readout processes of these multiplexing dimensions are achieved by independently controlling corresponding physical quantities.

The magnetic multiplexing dimension based on the Zeeman effect is illustrated in Figures 1c and S1a (Supporting Information). The Zeeman splitting degree of spin structures with different axial directions varies under the same magnetic field.^[^ ^12^, ^13^, ^14^ ^]^ The magnetic field intensity required for decoding codes in different axial directions also varies, enabling the construction of magnetic dimension based on the magnetic field (*M*) intensity. Two spin structures (AD_1_ and AD_2_) that only differ in axial direction are excited by a fixed frequency(*f*_fix_) microwave pulse MW_fix_ and subjected to a vertical magnetic field while keeping other conditions constant. Due to variations in components of the same magnetic field projected onto different spin axial directions, when AD_1_ spin structures resonate with the *M*_1_, AD_2_ spin structures resonate with *M*_2_ (Figure 1c). When applying *M*_1_, MW_fix_ induces a transition of AD_1_ from the |0> state to the |1> state (codes of 1), while AD_2_ remains in the |0> state. Similarly, when applying *M*_2_, AD_2_ is in the |1> state, while AD_1_ is in the |0> state. When decoding, |1> state (corresponds to code of 1) is read by the difference in PL intensity between it and |0> state (corresponds to code of 0). For the same one encoding unit, the integration of multiple spin structures with different axial directions enables the realization of multiple magnetic multiplexing channels. Code variations along the X, Y, and Z axis can be achieved by integrating spin structures with distinct axial directions in different encoding units within the same magnetic channel. In the magnetic dimension, each axial direction (AD_i_) corresponds to a resonant magnetic field *M*_i_ that induces a spin state transformation to |1>. Each green arrow in lower dashboards of Figure 1c represents an axial direction (AD_i_).

The wavelength multiplexing dimension based on PL is shown in Figures 1d and S1b (Supporting Information). The wavelength multiplexing channels can be constructed by different PL peaks^[^ ^12^, ^13^, ^17^, ^18^, ^19^, ^20^, ^21^ ^]^ of a variety of color centers in SiC. The same microwave excitation was applied to two spin structures (CC_1_, CC_2_) that only differ in color center types in order to induce both transitions to |1> state. The PL was passed through a filter, while all other conditions remained unchanged. The PL wavelengths of CC_1_ and CC_2_ are *λ*_1_ and *λ*_2_ respectively, due to the variation in the forbidden bandwidth among different color centers. (Figure 1d). Only the CC_1_ can be read when the filter passband contains *λ*_1_ exclusively, with the codes for CC_1_ and CC_2_ being 1 and 0 respectively. Similarly, when the filter passband exclusively contains *λ*_2_, only the CC_2_ can be read, with the codes of CC_1_ and CC_2_ being 0 and 1. Similar to magnetic dimension, multiple wavelength channels of the same encoding unit are achieved by integrating multiple types of color centers, and the variations of codes in the X, Y, and Z are achieved through the distinct types of color centers in different encoding units. In the wavelength dimension, each color center CC_i_ corresponds to a PL wavelength *λ*_i_, and corresponding code is 1. Each red arrow in lower dashboards of Figure 1d represents a color center (CC_i_).

The microwave multiplexing dimension based on the Rabi oscillation is shown in Figures 1e and S1c (Supporting Information). The Rabi frequency is directly proportional to the square root of microwave power,^[^ ^12^, ^17^, ^23^, ^24^ ^]^ and the multiplexing channel can be constructed by controlling the spin state based on the microwave pulse duration time. When the microwave pulse with a power gradient is applied to two spin structures (H_1_, H_2_) that only differ in spatial heights, while other conditions are kept consistent. The microwave powers received by H_1_ and H_2_ are *P*_1_ and *P*_2_ respectively, and the corresponding Rabi frequencies are Ω_1_ and Ω_2_ respectively. The duration of the microwave pulse required to fully transition the spin states of H_1_ and H_2_ from |0> to |1> is *t*_1_ and *t*_2_ respectively (Figure 1e). By designing the height of H_1_, H_2_, so that Ω_1_ = 2^k^·Ω_2_, following a microwave pulse with duration *t*_1_, H_1_ undergoes a complete transition from |0> state to |1> state, while H_2_ remains in |0> state with an incomplete transition. After a microwave pulse of duration *t*_2_, H_2_ undergoes a complete transition from |0> state to |1> state, while H_1_ transitions back to |0> state. The integration of multiple spin structures with varying heights within a single encoding unit enables the realization of microwave channels. Code variations along X, Y, and Z can be achieved by spin structures with distinct heights in different encoding units. In the microwave multiplexing dimension, each spin structure height H_i_ corresponds to a microwave pulse duration *t*_i_ that completely transaction it to |1> state. Each blue arrow in lower dashboards of Figure 1e represents a height (H_i_). Detailed spin state control scheme and multiplexing design are presented in Note S1 (Supporting Information).

The polarization multiplexing dimension based on dipole transition is shown in Figures 1f and S1d (Supporting Information). The spin structures of SiC color centers have multiple dipole directions, corresponding to various polarization directions of excited light,^[^ ^18^, ^19^, ^26^, ^27^, ^28^ ^]^ which can be used to construct polarization multiplexing channels. If the same microwave excitation is applied to two spin structures (DD_1_, DD_2_) that only differ in dipole direction so that all transition to |1> state. The PL with specific polarization is collected using a polarizer while maintaining other conditions consistent. The PL polarization of DD_1_ and DD_2_ are *θ*_1_ and *θ*_2_ respectively due to the presence of different dipole directions (Figure 1f). When only the PL with polarization direction *θ*_1_ is collected, the code of DD_1_ is 1 and that of DD_2_ is 0. Correspondingly, when only the PL with polarization direction *θ*_2_ is collected, the code of DD_1_ is 0 and that of DD_2_ is 1. Similar to magnetic multiplexing dimension, by integrating multiple spin structures with various dipole directions in the same encoding unit and spin structures with different dipole directions in different encoding units, multiple polarization channels and code variations along X, Y, and Z can be achieved by spin structures with distinct dipole directions in different encoding units respectively. In the polarization multiplexing dimension, each dipole directions DD_i_ corresponds to a polarization *θ*_i_ with a code of 1. Each yellow arrow in lower dashboards of Figure 1f represents a dipole direction (DD_i_).

In summary, the 7D multiplexing based on spin structures is achieved by controlling the magnetic field, wavelength, microwave pulse duration, and polarization to read the axial direction, type, height, and dipole direction (the hollow arrows in Figure 1g) of spin structure respectively. The solid arrows in the lower dashboard depict the applied physical quantity. When the solid arrow coincides with a hollow arrow, the applied physical quantity is consistent with the physical quantity required for code of 1, otherwise the code is 0. The aforementioned method enables the encoding of various images, such as “N”, “U”, “C”, and others, within the device.

## 3 Implementation and Multiplexing Capability of Spin‐Multiplexing

The implementation of SMED with 7 multiplexing dimensions is shown in Figures 1b and S1e (Supporting Information), spin structures of various physical properties (axial, type, height, and dipole direction) are prepared in each encoding unit. The encoding unit is subjected to a uniform magnetic field with an intensity of *M*_i_ in the +Z direction. A 532 nm laser is utilized for pumping the spin structures. A microwave pulse MW_fix_ with frequency *f*_fix_, power gradient along the +Z direction, and duration *t*_i_, is applied to induce transitions between spin states. Finally, the PL emitted by spin structures is collected after passing through a filter with a central wavelength *λ*_i_ and a polarizer with polarization *θ*_i_. The code of spin structures with axial direction AD_i_, type CC_i_, height H_i_, and dipole direction DD_i_ can be obtained by controlling four parameters. For three dimensions of space, the SMED is composed of encoding units distributed in the X and Y axis. Multiplexing in the Z‐axis is achieved by employing multiple layers of SMED in the Z‐direction, with an interlayer distance that exceeds the thickness of SMED. The 7‐D Spin‐multiplexing is ultimately realized through the combination of four additional dimensions and three spatial dimensions.

The multiplexing ability of SMED depends on both its spectral characteristics and physical structure (detail in Note S2 and Figure S2, Supporting Information). In terms of spectral lines, the number of multiplexing channels is determined by the range of available values and the linewidth, i.e., *N*_dimension_ = *R*_pq_/*FWHM*_pq_. As shown in Figure S2a (Supporting Information), the number of channels in the magnetic dimension depends on the spin‐magnetic response rage and the peak linewidth of magnetic resonance. Considering the angle difference of axial direction Δ*θ* shown in Figure S2b (Supporting Information), the number of channels in magnetic dimension can reach up to 270. For wavelength dimension, because of the available wavelength range (≈1.1 µm) and narrow peak width (≈1.1 nm) of PL, the number of channels is mainly determined by the number of spin structure types. Thereby the number of channels of wavelength dimension can reach 92 (Figure S2c,d and Table S1, Supporting Information). For microwave dimension, considering the wide available time duration range (≈103 s), ultra‐short oscillation period (≈1 ns), and minimum height difference *ΔH* of spin structures while manufacturing, the number of channels in microwave dimension is up to 36 (Figure S2e,f, Supporting Information). For polarization dimension, the number of channels mainly depends on the number of polarization angles of spin structure PL and is up to 11(Figure S2g,h, Supporting Information). Therefore, Spin‐multiplexing has an extremely large number of multiplexing channels in each dimension, i.e., an extremely high information density. The specific calculation process and discussion of multiplexing channels are shown in the Experimental Section and Note S3 (Supporting Information). The above address method is based on the mature PL difference readout method, so the improvement of spin multiplexing capability depends on the development of solid‐state spin systems with larger PL differences. Alleviating spin multiplexing's dependence on PL differences by developing new address methods will help to further break spin multiplexing's storage limitations (detailed analysis is described in Note S3, Supporting Information).

The detailed implementation condition of Spin‐multiplexing is shown in the upper part of Figure S1e (Supporting Information). The above methods enable SMED to achieve 7 multiplexing dimensions, which is the largest number of dimensions reported so far as we know (Figure S1f, Supporting Information). In terms of the number of multiplexing channels, optical multiplexing has recently achieved a breakthrough in the theoretical upper limit of the number of channels in the polarization dimension. However, there is no apparent theoretical upper limit for the number of channels in the four dimensions of SMED, as depicted in the lower part of Figure S1f (Supporting Information), indicating the potential to exploit multiplexing dimensions through a novel design paradigm utilizing fundamental physical effects.

## 4 Preparation and Characterization of SMED

Conventional solid‐state spin systems hinder the achievement of Spin‐multiplexing due to the lack of rich spin properties (orientation, form, and spatial distribution of crystal). In order to prepare spin structures based on SiC with rich axial directions, types, spatial heights, and dipole directions, we designed a laser direct writing scheme to convert Polydimethylsiloxane‐polyvinyl chloride (PDMS‐PVC) into SiC.^[^ ^6^, ^30^, ^31^, ^35^, ^36^, ^38^ ^]^ Pure PDMS is a transparent flexible substrate, and the SiC synthesized through one‐step laser direct writing on pure PDMS exhibits a low yield and a restricted distribution.^[^ ^35^ ^]^ Therefore, through precise control over the laser direct writing method, reaction environment,^[^ ^30^ ^]^ and precursor material,^[^ ^30^, ^34^, ^36^ ^]^ we have successfully achieved high‐yield fabrication^[^ ^6^, ^36^ ^]^ of SiC with abundant attributes, widespread distribution, and controllable size.

**Figure 2:**
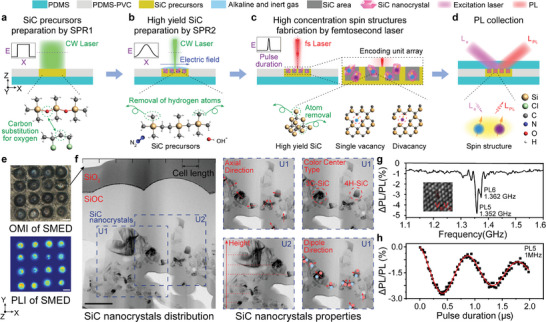
Fabrication and product characterization Spin‐multiplexing. a) Preparation of SiC precursors (PCS and PCS‐C) from PDMS‐PVC by using a flat‐top laser (SPR1). b) SiC preparation from SiC precursors by focused Gaussian laser (SPR2). c) SiC spin structures fabrication by femtosecond laser. d) PDMS protective layer fabrication and PL acquisition. The insets in a and b are the energy (E) distribution of the laser beam on the X‐axis (purple dash line). The inset in c is the energy (E) related to the pulse duration of the fs laser (purple dash line). The main two kinds of SiC spin structures that fabricated by femtosecond laser. e) Optical microscopic image (OMI) and PL image (PLI) of SMED_4 × 4_. f) TEM image (left part) and properties analysis (right part) of SiC nanocrystals on the cross‐section (scale bar, 500 nm). Black regions are SiC nanocrystals. The axial direction is perpendicular to the direction of crystal growth, and the axial direction of the twin crystal is marked by the vector sum of the axial direction of each single crystal. The dipole direction is calibrated by the direction of the crystal in the plane that is perpendicular to the axial direction. g) Continuous‐wave ODMR spectrum of PL5 and PL6 in 4H‐SiC. PL5 and PL6 peaks are at 1.352 and 1.362 GHz, respectively. The inset shows the TEM image of divacancy (V_Si_V_C_), and the high‐resolution TEM images of color centers are shown in Figure S5f,g (Supporting Information). h) Rabi oscillation of PL5 in Spin‐multiplexing.

The fabrication of SiC is shown in **Figure** **2**a–d. To improve the yield of SiC, polyvinyl chloride (PVC) was selected as the carbon source for the elimination reaction, and the unsaturated carbon produced replaced the oxygen atoms in the molecular frame structure of PDMS (O─Si─O).^[^ ^30^, ^34^, ^37^ ^]^ The high‐yield SiC nanocrystals are fabricated through three laser direct writings on the PDMS‐PVC mixture after beam shaping. The first two processes are continuous‐wave laser direct writing, wherein the mixture is subjected to temperatures of ≈450 °C and >1000 °C respectively, inducing two selective pyrolysis and recombination (SPR1, SPR2). During SPR1, the excellent precursors (polycarbosilane (PCS) and polycarbosilane‐carbon (PCS‐C)) of SiC were prepared by co‐pyrolysis of PDMS and PVC. Through SPR2, SiC nanocrystals are prepared at a high yield benefiting from its excellent precursors, inert gas protection, regulation of free radicals, and appropriate pyrolysis parameters. After characterizing the crystal structure, form, and orientation of each region using X‐ray diffraction (XRD),^[^ ^39^, ^40^, ^41^, ^42^, ^43^ ^]^ the desired spin structures are prepared in specific SiC nanocrystals through femtosecond laser direct writing.^[^ ^28^, ^31^, ^32^, ^33^ ^]^ The concentration, type, and height of the spin structures were precisely controlled by manipulating the pulse energy and focal plane height. As shown in Figure 2a–e, by this method, we selectively prepare spin structures with extremely rich axial directions, types, heights, and dipole directions, and prepare a 4 × 4 encoding unit array (SMED_4 × 4_). The regions with required spin structures are determined through analysis of optical detection magnetic resonance (ODMR), Rabi, PL, and polarization spectra. These essential regions are retained while any unnecessary portions are removed by using a focused ion beam (FIB) or other appropriate techniques. Finally, a PDMS protective layer is applied to the aforementioned material to create a fully‐formed SMED. Details of preparation and operation are provided in Note S4 (Supporting Information).

The prepared SMED by the above process has good flexibility and transparency (Figure S5a, Supporting Information). Transmission electron microscope (TEM) images^[^ ^44^ ^]^ and selected area electron diffraction (SAED) analysis^[^ ^44^ ^]^ prove the rich orientations, types, and heights of SiC nanocrystals (Figure 2f; Figure S5d,e, Supporting Information). The single vacancy and divacancy can be visually observed by magnifying the high‐resolution TEM images (Figure S5f,g, Supporting Information, and the inset of Figure 2g). At the same time, the PL and magnetic resonance effect of the spin structures are verified by ODMR, such as PL5, and PL6 (Figure 2g; Figure S6a,b, Supporting Information). The spin state control system of the microwave pulse sequence is used to characterize the Rabi oscillation of PL6, with oscillation frequencies at ≈1 and ≈4 MHz respectively (Figure 2h; Figure S6c, Supporting Information). The aforementioned systems provide the equipment foundation for constructing four additional dimensions based on spin structures.

## 5 Encoding Verification of 7D Spin‐Multiplexing

**Figure 3:**
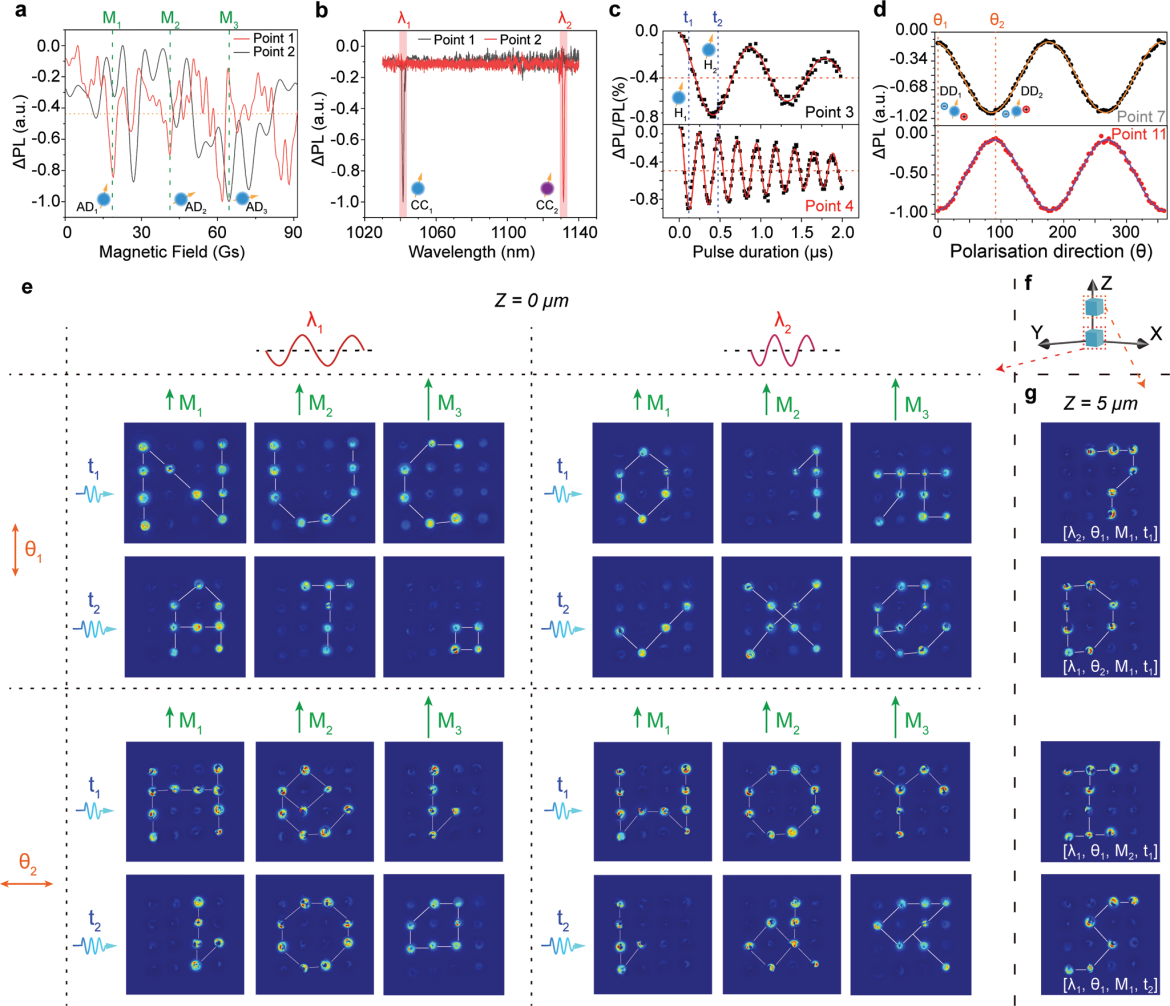
Encoding mechanism and corresponding code images of Spin‐multiplexing. a–d) Spectra used for encoding of magnetic field dimension (a), wavelength dimension (b), microwave dimension (c), and polarization dimension (d). The spectra show different peak positions due to the different spin properties of each point. The 16 points are labeled as Point 1 to Point 16 from left to right and from top to bottom. e) Code images of the Z = 0 µm plane. A total of 24 multiplexing images are encoded in three magnetic field channels (*M*_1_‐*M*_3_), two wavelength channels (*λ*_1_, *λ*_2_), two microwave channels (*t*_1_, *t*_2_), and two polarization channels (*θ*_1_, *θ*_2_). Encoding units with bright points possess spin structures with the corresponding properties required for each multiplexing channel, while dark spots do not. Therefore, bright points represent the code 1, and dark points represent the code 0. f) Schematic diagram of multiplexing in Z direction. Above and below cubes represent the Spin‐multiplexing structures in *Z* = 5 µm and *Z* = 0 µm planes, respectively. g) Code images of the *Z* = 5 µm plane.

Based on SMED_4 × 4_, we first verify the multiplexing ability and performance of SMED on seven dimensions. The codes of SMED are defined and designed based on quantum states of spin structures. To decode these codes, we employ the collection of different images in PL intensity, which is the codes and represents the corresponding quantum states. The difference images are obtained by calculating the absolute value of the difference between PL image of each multiplexing channel ([*λ*_m_, *θ*_n_, *M*_p_, *t*_q_]) and the initial PL image. The initial PL images are acquired without microwave pulse, specifically when all spin structures are in the |0> state (Experimental Section; Note S4, Supporting Information). For spin structures that only differ in axial direction, when applying *M*_i_, the code of units containing AD_i_ is 1, and code of other units is 0. For spin structures that only differ in type, when the passband only contains *λ*_i_, the code of units containing CC_i_ is 1, and code of other units is 0. For spin structures that only differ in height, when the duration is *t*_i_, the code of units containing H_i_ is 1, and the code of other units is 0. For spin structures that only differ in dipole direction, when the polarization direction is *θ*_i_, the code of units containing DD_i_ is 1, and the code of other units is 0 (**Figure** **3**a–d; Figures S9–S12, Supporting Information). In theory, the five additional dimensions and their multiplexing channels are independent of each other. However, due to the appearance of non‐idealized control devices and redundant spin structures, the above independence is weakened. Therefore, optimizing the control device and removing the redundant spin structure will help to further improve the independence between the dimensions and the channels (Note S5, Supporting Information).

As the decoding result, the difference images of PL intensity of SMED are shown in Figure 3e. In the 120 × 120µm^2^ area of the SMED_4 × 4_, 24 multiplexing channels with corresponding codes were achieved through the utilization of three magnetic field strengths (*M*_1_ = 18.5 Gs; *M*_2_ = 41.2 Gs; *M*_3_ = 64.5 Gs), two wavelengths (*λ*_1_ = 1042.7 nm; *λ*_2_ = 1131.5 nm), two microwave duration (*t*_1_ = 118 ns; *t*_2_ = 475 ns) and two polarization (*θ*_1_ = 0°; *θ*_2_ = 90°). On the plane of Z = 0 µm, the letters “N”, “U”, and “C” were encoded into the channels [*λ*_1_, *θ*_1_, *M*_1_, *t*_1_], [*λ*_1_, *θ*_1_, *M*_2_, *t*_1_], and [*λ*_1_, *θ*_1_, *M*_3_, *t*_1_] respectively. Furthermore, various numbers, special characters and letters were also encoded into other channels in four additional multiplexing dimensions. The outstanding multiplexing encoding capability of the SMED in four additional dimensions has been validated.

In addition, the multiplexing ability of SMED in Z‐axis has been verified. On the plane of *Z* = 5 µm, four channels are selected for encoding (Figure 3g), and the corresponding patterns are obtained: “7′, ‘D”, “I”, and “S” (7‐D Information Storage). According to the above results, the test results are completely consistent with the coding and writing results, whether it is letters, numbers or graphic information, which verifies the 7‐D multiplexing ability of SMED. According to the above results, the readout results are completely consistent with the encoding images, whether it is letters, numbers, or special characters, which verifies the encoding accuracy of SMED in 7 dimensions. The corresponding data and analysis are described in Note S5 (Supporting Information), and the digitized codes are shown in Figure S13 and Note S6 (Supporting Information).

Due to the wide value range of channels and narrow channel width, SMED has a large number of channels without a theoretical upper limit (Note S4, Supporting Information). The number of channels is currently constrained by the existing signal‐to‐noise ratio of PL signals, but it is expected to significantly increase in the future as the signal‐to‐noise ratio continues to improve. The four physical quantities of SMED are fully electrically controlled, thereby enhancing the flexibility of multiplexing channels and enabling the configuration of more complex and diverse dynamic encoding. This significantly enhances the ability of SMED in real‐time storage, anti‐counterfeiting, and encryption of vast amounts of information (Note S8, Supporting Information).

## 6 Real‐Time and Highly Dynamic Encryption/Watermarking

In addition to necessitating substantial capacity and high information density of equipment, the storage/encryption of massive information also demands fast processing speed and exceptional dynamic performance. For instance, agents must quickly capture photographs of massive confidential documents and transmit them after real‐time encryption/watermarking. The managers are required to apply marks/watermarks to the electronic archiving of massive paper archives, which necessitates the utilization of high‐speed equipment for real‐time and efficient marking/watermarking, as well as archiving/transmission.

**Figure 4:**
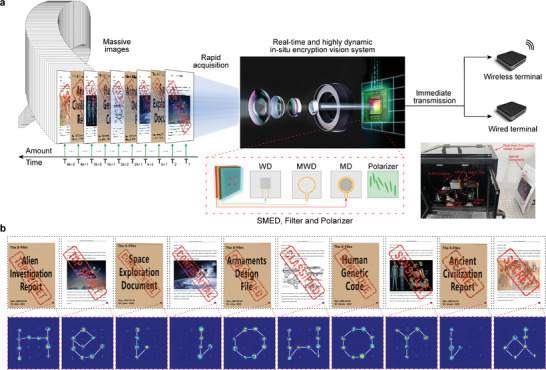
Real‐time and highly dynamic encryption/watermarking system based on SMED. a) The schematic diagram of system composition and system application scheme. The inset at the bottom right shows the encryption/watermarking system and encrypted/watermarked objects (secret document). The system consists of a laser, two polarization rotators (PR), a control chip, a lens, and a camera with SMED. b) Imaging results of secret document encryption. The images in the first row are whole encrypted images, and the images in the second row are enlarged images of the SMED region. White lines in second‐row images are additional lines added to aid reading.

To this end, we demonstrate a practical process based on a vision system integrated with SMED for the encryption/watermarking of massive X‐files during their collection and immediate archiving/transmission. Based on the spin physical effects, the free transformation of spin state was regulated as the dynamics multiplexing mode into high dynamic applications. As shown in **Figure** **4**a, detailed equipment information is described in Note S7 (Supporting Information). A vast collection of X‐files (including alien archives, space exploration archives, design drawings of military equipment, human genetic code archives, ancient civilization books, etc.) is systematically acquired and inputted into the vision system at high frequency. The SMED is integrated with imaging devices to construct an in situ encryption/watermarking device for optical images. The SMED generates dynamic keys to real‐time encrypt/watermark the collected images at high frequency. The resulting ciphertext or marked images can be promptly transmitted to the distribution device, and subsequently distributed and transmitted to each terminal through wired or wireless means.

First, we prove the excellent dynamic performance of SMED. As the magnetic field, microwave, tunable filter, and polarizer are all controlled by voltage or current devices, SMED has the expected high dynamic frequency and accuracy. The investigation of the relationship between bit error rate and dynamic frequency in four dimensions reveals that, under a 2% tolerance for bit error rate, the dynamic frequency of SMED can reach up to 500 kHz. This effectively enhances the dynamic capacity of multiplexing technology (Figure S5 and Note S8, Supporting Information).

Based on the in situ encryption/watermarking system shown in the inset of Figure 4a, a dynamic time sequence was constructed by utilizing multiplexing channels encoded as “h”, “e”, “l”, “l”, “o” and “w”, “o”, “r”, “l”, “d” respectively (Figure 4b) with a sequence in Figure S15c (Supporting Information). When the graphic information of the X‐files was collected by the vision system at a frequency of 50 Hz, SMED generated keys at a frequency of 50 Hz to encrypt/watermark the graphic information. As illustrated in Figure 4b, it is evident that the captured images have been promptly encrypted/watermarked by SMED in real‐time, with clearly visible codes of “h”, “e”, “l”, “l”, “o”, “w”, “o”, “r”, “l”, “d”, thereby validating the practical application of SMED within the in situ encryption/watermarking vision system. The above demonstration is based on the magnetic field, wavelength, and microwave duration time dimensions with fast response speeds. Due to the slow response speed of polarization and Z dimensions, some image frames are not encrypted (Figure S15d, Supporting Information). Therefore, polarization and the Z dimension are not suitable for the above application scenarios.

Moreover, SMED can also be applied to traditional encryption methods and anti‐counterfeit labels. The specific implementation method is detailed in Note S7 (Supporting Information). The 24 multiplexing channels are utilized for constructing a dynamic time sequence. To ensure a bit error rate <0.9%, the dynamic frequency is set at 1 kHz. Even with a high‐speed computer attempting 10^14^ tries/s, cracking the time sequence still requires an astonishing duration of ≈1.33 × 10^11^ years when the sequence length exceeds 24 ms (Figure S15a, Supporting Information). The cracking of the dynamic sequence of SMED would demand nearly the entire lifespan of the universe (≈1.4 × 10^11^ years). Therefore, it is anticipated that SMED will unlock the eternal door of encryption technology.

## 7 Conclusion

This paper presents a 7‐D multiplex technology. By controlling the magnetic field, wavelength, microwave pulse duration, and polarization respectively, the codes of axial direction, types, spatial height, and polarization are extracted based on the corresponding physical effects. The corresponding number of multiplexing dimensions reaches 7 (contains three dimensions of space), which is >6dimensions in the previous study. Moreover, the design of multiplexing dimensions from the fundamental physical effects breaks the theoretical limitation of physical structure parameters. As more physical effects of spin structures are discovered, the number of multiplexing dimensions will be infinite in the future. Additionally, based on the fully electronic control scheme, SMED offers a unique advantage with its ability to achieve dynamic frequencies up to 0.5 MHz. This feature is particularly beneficial for in situ real‐time encryption/watermarking and immediate transmission. Due to the flexibility, high transparency, easy integration, low‐cost, simple manufacturing, and fully electronic control scheme of SMED, it facilitates the realization of the engineering application of multiplex technology. Moreover, the in situ encryption/watermarking system for images encrypts/watermarks images without relying on electronic computing devices, thereby preventing side‐channel attacks on the electronic system and enhancing security.^[^ ^45^, ^46^ ^]^ This approach provides a new paradigm for advancing the next generation of multiplexing technology.

## 8 Experimental Section

### Materials and Systems for Spin Structure Fabrication

The fabrication of SiC precursor (PDMS‐PVC) consists of five steps: mixing, stirring, spin coating, removing bubbles, and curing. PDMS‐PVC mixture was prepared by mixing PDMS (Sylgard 184, from Dow Corning) and PVC (PVC S‐700, from Sinopec). After mixing the PDMS‐PVC mixture thoroughly with a magnetic stirrer (MS‐H‐ProT, from Scilogex), the mixture was spin‐coated into a thin film in a petri dish using a spin coater. The bubbles in the mixture film were removed by placing the film in vacuum drying oven (PVD‐1000, from Shanghai SHIBEI) for 1 h. Subsequently, the mixture film was transferred to the heating platform (80 °C for 25 min) for constant temperature annealing for curing. The spin structures fabrication system mainly consists of continuous wave (CW) laser, femtosecond laser, microscope, laser beam shaper, gas chamber, and precision displacement table. CW laser (MW‐GL‐532/2000 mW, from Changchun Laser Optoelectronics Technology Co.) was used for preparing SiC during SPR1 and SPR2, and beam shaper (FLE11, from Thorlabs) could reshape the Gaussian laser beam into a flat‐top laser. Femtosecond laser (CARBIDE CB5‐SP, from Light Conversion) was used to fabricate spin structures in SiC. The protection of the SiC was achieved by placing its precursors in a self‐developed gas chamber filled with an inert gas. After laser was applied to the surface of the precursors through a microscope (BX53M, from Olympus), silicon carbide and its spin structure were prepared. And the microscope was also used to observe and analyze the prepared products. Moreover, the electronically controlled precision displacement table (PTS‐50‐25‐2G, from Sanying Motion Control Instruments Ltd.) ensures the precision of SiC fabrication and spin structure fabrication.

Details of the fabrication process are described in Note S4 (Supporting Information).

### Characterization of SiC and Spin Structures

The characterization of SiC includes microscopic image, Raman spectrum, XRD, TEM, SAED, ODMR, and Rabi oscillation. The microscopic images were obtained by the same microscopic system used in the preparation process. The Raman spectra measured by Renishaw invia were used to analyze the product distribution. The spectra of micro‐area XRD (Ultima IV, from Rigaku) were used to analyze the crystal form and orientation. TEM and SAED (Tecnai G2 20, from FEI Company) were used to analyze the properties of SiC crystals with Wintech‐nano assistance. The ODMR spectra and Rabi oscillation signals were collected by a photodetector (APD430C/M, from Thorlabs). During collecting the ODMR spectra and Rabi oscillation signals, the microwave pulses supplied by an arbitrary waveform generator (AWG5204, from Tektronix) were radiated to the SMED by a circular printed circuit board (PCB) antenna, and near infrared laser (MW‐RIR‐915/50 mW, from Changchun Laser Optoelectronics Technology Co.) excited the spin structures after passing through the near infrared objective lens (Plan Apo NIR 50X/0.42, from Mitutoyo). Moreover, the PL images were acquired infrared CCD camera (Wildcat+ 1280, from Xenics) under the same acquisition conditions as ODMR and Rabi.

Details of the characterization are described in Notes S4 and S5 (Supporting Information).

### PL Image Acquisition System and Analysis Method

Under the control of self‐developed physical quantity control device, the PL images were captured by the infrared CCD camera while maintaining the same excitation laser and microwave as during Rabi signal acquisition. The PL difference images were obtained by differentiating two PL images by Matlab 2020b. Details of the characterization are described in Notes S5–S7 (Supporting Information).

## Conflict of Interest

The authors declare no conflict of interest.

## Author Contributions

Y.Q., H.G., and J.T. performed conceptualization; Y.Q., H.G., J.T., H.T., Z.L., H.W., Z.M. and X.L. performed methodology; Y.Q. and H.G. performed investigation; Y.Q., H.G., M.X., X.Y., J.Q. and P.Z. performed visualization; H.G. and J.L. performed funding acquisition; H.G. and J.L. performed project administration; H.G. and J.L. performed supervision; Y.Q. and H.G. wrote the original draft and the rest of the authors; Y.Q. and H.G. Wrote and reviewed and performed editing and the rest of the authors. Y.Q. and H.G. contributed equally to this work.

## Supporting information

Supporting Information

Supplemental Movie 1

Supplemental Movie 2

## Data Availability

The data that support the findings of this study are available from the corresponding author upon reasonable request.
