# Supplementary material for: 7D High‐Dynamic Spin‐Multiplexing
Source: Adv Sci (Weinh). 2024 Jun 28;11(33):2402378. doi: 10.1002/advs.202402378 (PMC11434207; doi:10.1002/advs.202402378)
Supplement: Supplementary file 1 — Supporting Information [file ADVS-11-2402378-s001.docx]

Supporting Information

Seven-dimensional high-dynamic Spin-multiplexing

Yue Qin †, Hao Guo †*****, Sebastian Pazos, Mengzhen Xu, Xiaobing Yan, Jianzhong Qiao, Jia Wang, Peng Zhou, Yang Chai, Weida Hu, Zhengqiang Zhu, Zhonghao Li, Huanfei Wen, Zongmin Ma, Xin Li, Mario Lanza*, Jun Tang*, He Tian*, Jun Liu*

Supplementary Text

**Note S1 Multiplexing dimension design and construction**

In the magnetic multiplexing dimension, the encoding process is achieved by controlling the axial directions of spin structures, and the codes is read by controlling the magnetic field intensity. The microwave MW_fix_ with a frequency of *f_fix_* is applied to spin structures, thereby the resonance between spin structure and MW_fix_ occurs with a vertical magnetic field of *M_fix_*. When the spin structures only differ in axial direction, each axial direction AD_i_ corresponds to a vertical magnetic field *M_i_* required for resonance. The encoded information of axial AD_i_ can be read by controlling the magnetic field intensity *M_i_*, once the wavelength, microwave pulse duration, and polarization have been determined (**Figure S1a**).

In the wavelength dimension, the encoding is realized by controlling the spin types and the codes are read by controlling the filter wavelength. When the axial direction, height and dipole direction are the same, and only the spin structure type is different, the energy level structure of the spin structure is quite different. The forbidden bandwidth and energy level difference between ground state and excited state is determined by the color center type CC_i_, and each CC_i_ corresponds to a luminescence wavelength. The codes of CC_i_ can be read through the filter wavelength *λ_i_* (**Figure S1b**).

In the microwave dimension, the encoding is achieved by controlling the heights of spin structures. A microstrip antenna with a certain microwave power gradient in the Z direction is used to encode the codes of heights by controlling the microwave pulse duration. When the axial direction, type, and dipole direction remain constant, but only the height of the spin structure varies, there is a variation in microwave power surrounding the spin structures, resulting in different Rabi oscillation frequencies. The code corresponding to the spin structure with a height of H_i_ can be read by applying a microwave pulse with the duration of *t_i_*, when the adjacent Rabi oscillation frequencies are in a multiple relationship (**Figure S1c**).

In the polarization dimension, the codes are encoded by controlling the dipole direction of the spin structure, and the codes are decoded by controlling the polarization direction of the PL. When the axial direction, type and height are the same, and only the dipole direction is different, the polarization of the PL is different. When the polarizer only supports the passage of light in one polarization direction of *θ_i_*, the codes of a single dipole direction (DD_i_) can be read (**Figure S1d**).


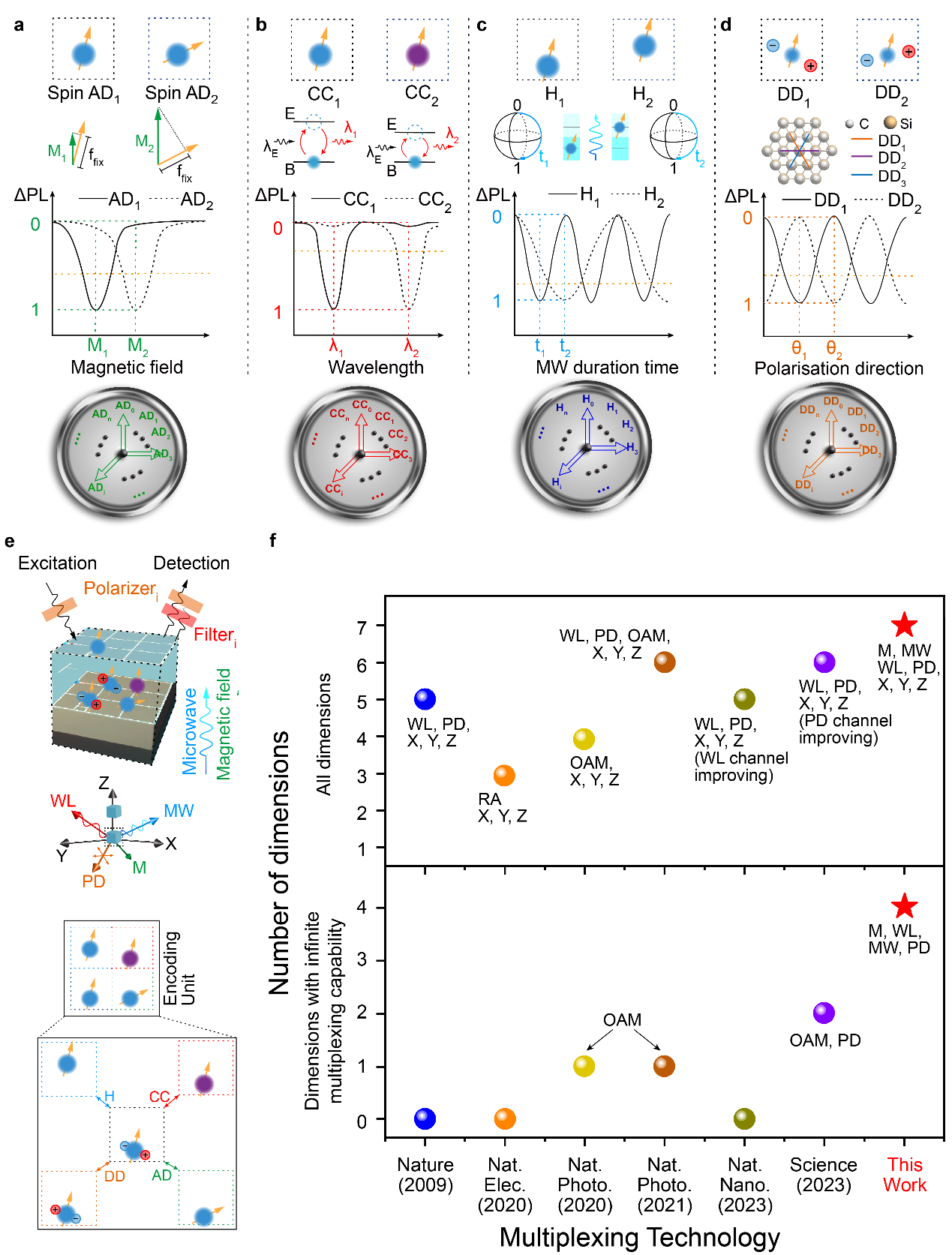


**Figure S1** **Mechanism of Spin-multiplexing and comparison of Spin-multiplexing and conventional multiplexing.** **a**-**d,** Design of spin multiplexing in the magnetic field dimension (a), wavelength dimension (b), microwave pulse duration dimension (c), and polarization dimension (d). **e**, Internal structure of Spin-multiplexing encoding unit (upper part), and reading process of codes (lower part). In addition to the three dimensions of space (X, Y and Z), the 7D Spin-multiplexing possess four additional dimensions (M (magnetic dimension), WL (wavelength dimension), MW (microwave dimension) and PD (polarization direction dimension)). **f**, Comparison of multiplex technology in number of dimensions.

**Note S2 Multiplexing capabilities (number of channels) of SMED**

The multiplexing ability of SMED relies on the spectral and physical properties of spin structures (**Figure S2**). The number of multiplexing channels in spectral properties for the magnetic field, wavelength, microwave pulse duration and polarization dimension of SMED are *MC_MS_*, *MC_WLS_*, *MC_MWS_* and *MC_PDS_*, respectively. They can be described by the following formula (**Figure S2**):

$$\begin{aligned} {MC}_{MS}=\frac{AMR}{{FWHM}_{M}}\#\left( 1 \right) \end{aligned}$$

$$\begin{aligned} {MC}_{WLS}=\frac{AWLR}{{FWHM}_{WL}}\#\left( 2 \right) \end{aligned}$$

$$\begin{aligned} {MC}_{MWS}=\log_{2} \frac{ATR}{T_{min}+1}\#\left( 3 \right) \end{aligned}$$

$$\begin{aligned} {MC}_{PDS}=\frac{APDR}{{FWHM}_{PD}}\#\left( 4 \right) \end{aligned}$$

*AMR*, *AWLR*, *ATR* and *APDR* are available magnetic field range, available wavelength range, available microwave pulse duration time range and available polarization direction range respectively; *FWHM_M_*, *FWHM_WL_* and *FWHM_PD_* are the full width at half maximum of the magnetic resonance peaks, and the PL peaks and polarization peaks respectively; *T_min_* is the minimum microwave pulse duration time.

The number of multiplexing channels in terms of physical properties of magnetic field, wavelength, microwave and polarization dimension of SMED are *MC_MP_*, *MC_WLP_*, *MC_MWP_* and *MC_PDP_*, respectively. And they can be determined by the following formula: (**Figure S2**):

$$\begin{aligned} {MC}_{MP}=\frac{\omega_{max}}{{\Delta\omega}_{min}}\#\left( 5 \right) \end{aligned}$$

$$\begin{aligned} {MC}_{WLP}=ACC\#\left( 6 \right) \end{aligned}$$

$$\begin{aligned} {MC}_{MWP}=\frac{H_{max}}{{\Delta H}_{min}}\#\left( 7 \right) \end{aligned}$$

$$\begin{aligned} {MC}_{PDP}=APD\#\left( 8 \right) \end{aligned}$$

*ω_max_*, *ACC*, *H_max_* and *APD* are maximum range of axial angle, number of available color center types, maximum range of height (usually is microwave pulse radiation range) and number of available dipole directions, respectively. Δ*ω_min_* and Δ*H_min_* are minimum axial angle difference and minimum height difference, respectively. In SMED, the number of multiplexing channels in each dimension is the minimum value between number of spectral multiplexing channels and the number of physical multiplexing channels, as follows:

$$\begin{aligned} {MC}_{M}=\min\left\{ {MC}_{MS},{MC}_{MP} \right\}\#\left( 9 \right) \end{aligned}$$

$$\begin{aligned} {MC}_{WL}=\min\left\{ {MC}_{WLS},{MC}_{WLP} \right\}\#\left( 10 \right) \end{aligned}$$

$$\begin{aligned} {MC}_{MW}=\min\left\{ {MC}_{MWS},{MC}_{MWP} \right\}\#\left( 11 \right) \end{aligned}$$

$$\begin{aligned} {MC}_{PD}=\min\left\{ {MC}_{PDS},{MC}_{PDP} \right\}\#\left( 12 \right) \end{aligned}$$

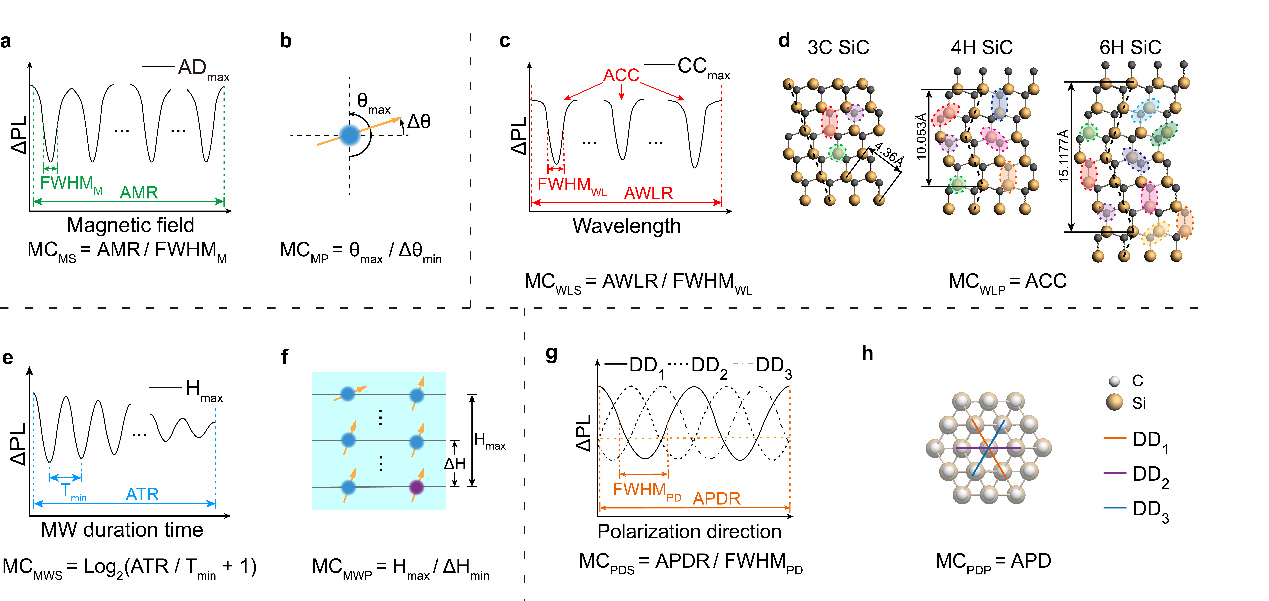


**Figure S2** Capabilities of SMED in the magnetic field dimension (**a**), wavelength dimension (**b**), microwave dimension (**c**) and polarization dimension (**d**).

**Note S3 Multiplexing capabilities of SMED with current technologies**

In summary, the multiplexing ability of SMED depends on the spectral and physical properties of spin structures (**Figure S2**). The number of multiplexing channels in each dimension depends on the available value range and the linewidth of each physical quantity, i.e., *N_dimension_*=*R_pq_*/*FWHM*_pq_. There are many channels in the magnetic field dimension, due to the narrow *FWHM_M_* (~3.57 Gs) of spin magnetic resonance peak, the wide available magnetic field range (*AMR*), (*AMR* is usually not clearly limited. If keep the m_s_=+1 and m_s_=-1 not mixed with each other, the maximum *AMR* can reach 964 Gs). *θ_max_* is usually 180°. The minimum angle difference is not limited by the manufacturing technology, Δ*θ_min_* can be very small, so it mainly depends on the minimum angle difference of crystal orientation that can be identified by XRD. Based on the current indexes of resonance peak linewidth and magnetic sensitivity, the number of channels of magnetic field dimension can reach *N_M_= AMR/FWHM*_M_=964/3.57=270. In terms of wavelength dimension, the number of channels is determined by spectral lines and color center types. In terms of spectral lines, the *FWHM_WL_* of PL peaks is as small as 1.1 nm, the *AWLR* can reach 1.1 μm. According to **Formula (2)** the number of channels can reach up to 1000. In terms of color center type, there are 52 kinds of SiC color centers reported so far (**Figure S2d**, **Table S1**). Therefore, the number of multiplexing channels in the wavelength dimension can reach at least 52 (**Formula (6)**). For all spin structures of color centers, the number of spin structure types is as high as 92. That means, if a hybrid SMED device based on diamond, SiC, BN, and other materials is established, the number of multiplexing channels in wavelength dimension can reach 92.

In terms of the microwave dimension, the Rabi oscillation can last for a long time under ideal conditions, with *ATR* up to 103s and the minimum oscillation period (*T_min_*) as low as 1ns. The height error of current color center manufacturing technology is ~100 nm, the microwave radiation distance of the microstrip antenna is on the order of millimeters, and there is no obvious limit on the number of multiplexing channels in terms of physical structure. Therefore, according to **Formula (3)**, the number of multiplexing channels that can be constructed is no less than 36. In terms of the polarization dimension, the polarization direction of the spin dipole corresponds to the polarization state of the emitted light, and the number of channels in the polarization dimension can be increased to 11 through the introduction of engineering noise. Therefore, each dimension of spin multiplexing has a very large number of multiplexing channels, as shown in **Figure S1f**.

PL difference has been proven to be a major, mature, stable, and convenient readout method for solid-state spin information, so we choose the address method based on the PL difference. The above address method makes the storage capacity of spin multiplexing depend on the total PL difference and the minimum PL difference that can be recognized. Therefore, when the above address method is adopted, the realization of high information density will depend on solid-state spin systems with large PL differences. The dependence on large PL differences limits the achievement and development of spin multiplexing in some solid-state spin systems, even though these solid-state spin systems exhibit excellent performance in other aspects such as wavelength and coherence time.

Therefore, the development of new address methods to alleviate or avoid the dependence on large PL differences will help spin multiplexing to be more widely implemented and have a larger storage capacity. In recent years, researchers have developed several new readout methods for solid-state spin systems, including spin-to-charge conversion, scanning tunneling microscopy (STM), and microwave resonators. Due to the high complexity, insufficient stability, high cost, and limited scope of implementation, the feasibility of the above address methods applied to spin multiplexing needs to be further improved. Specifically as follows: 1. The spin-to-charge conversion method has not been verified in broader solid-state spin systems (such as SiC color centers), and the ionization process is relatively complex and expensive; 2. The STM method requires a slow scan of the entire plane, so the readout time is very long. The STM is extremely expensive and large, so the cost is very high and the application spin system needs to be further developed; 3. The microwave resonator has a large volume and the applicable spin system needs to be further developed. Therefore, it is an important development direction to develop new and more effective address methods to solve the dependence of spin multiplexing on large PL differences.

**Table S1** Spin structures of color centers in SiC crystals

|  | Color centers | Abbreviations  used in literature | Zero-phonon line (nm) | Zero field splitting (GHz) |
| --- | --- | --- | --- | --- |
| 3C | Si_C_ | D_1_ | 628 |  |
|  | Si_C_C_Si_ | A_0_ | 648 |  |
|  | C_Si_V_C_ | E |  |  |
|  | V_Si_ | T1 |  |  |
|  | V_Si_V_C_ | RL1, Ky5 | 1107 | 1.328 |
|  | N_C_V_Si_ | NV | 1468 | 1.303 |
| 4H | V_Si_ | V1, V1’ | 862(V1), 858(V1’) | 0.070 |
|  |  | V2 | 917 | 0.004 |
|  | V_Si_V_C_ | PL1 | 1132 | 1.336 |
|  |  | PL2 | 1131 | 1.305 |
|  |  | PL3 | 1108 | 1.222 |
|  |  | PL4 | 1078 | 1.334 |
|  |  | PL5 | 1043 | 1.373 |
|  |  | PL6 | 1038 | 1.365 |
|  |  | PL7 |  |  |
|  |  | PL8 | 1007 | 1.3987 |
|  | N_C_V_Si_ | PLX1 | 1180 | 1.193 |
|  |  | PLX2 | 1223 | 1.282 |
|  |  | PLX3 | 1241 | 1.328 |
|  |  | PLX4 | 1242 | 1.331 |
|  | C_Si_V_C_ | A1, B1 | 648.5(A1), 671.6(B1) |  |
|  |  | A2, B2 | 651.7(A2),  672.85(B2) |  |
|  |  | B3 | 675.1 |  |
|  |  | B4 | 676.4 |  |
|  | Mo |  | 1076.2 | 3.3 |
|  | V^4+^ | V^4+^ (h) | 1278.808 | 529 |
|  |  | V^4+^ (k) | 1335.331 | 43 |
|  | Cr^4+^ | Cr_C_ | 1042 | 6.707 |
|  |  | Cr_A_ | 1070.3 | 1.0627 |
| 6H | V_Si_ | V1 | 865 | 0.00278 |
|  |  | V2 | 887 | 0.128 |
|  |  | V3 | 907 | 0.266 |
|  | V_Si_V_C_ | QL1 | 1140 | 1.300 |
|  |  | QL2 | 1136 | 1.334 |
|  |  | QL3 | 1124 | 1.236 |
|  |  | QL4 | 1108 | 1.317 |
|  |  | QL5 | 1093 |  |
|  |  | QL6 | 1093 | 1.347 |
|  |  | QL7 |  | 1.345 |
|  |  | QL8 |  | 1.371 |
|  |  | QL9 |  | 1.349 |
|  | N_C_V_Si_ |  | 1328 | 1.291 |
|  |  |  | 1278 | 1.305 |
|  |  |  | 1345 | 1.240 |
|  | C_Si_V_C_ | A1, B1 | 741.1(A1),  757.9(B1) |  |
|  |  | A2, B2 | 747.6(A2)  760.1(B2) |  |
|  |  | A3, B3 | 751.5(A3)  764.4(B3) |  |
|  |  | B4 | 768.9(B4) |  |
|  | Mo |  | 1121.3 | 3.3 |
|  | V^4+^ | V^4+^ (h) | 1308.592 | 524 |
|  |  | V^4+^ (k_1_) | 1351.845 | 25 |
|  |  | V^4+^ (k_2_) | 1387.806 | 16 |

**Note S4 Preparation and characterization of SMED**

The preparation process of SMED mainly consists of three steps: SPR1, SPR2 and spin structure preparation. First, PDMS and PVC were mixed in a ratio of 10:1, and the mixture was spin-coated on a cured PDMS substrate and held at 85°C for 2 hours to solidify the mixture (**Figure S3a**). The cured mixture was irradiated with a continuous-wave laser with a uniform energy distribution and heated to ~450 °C to cause the first pyrolysis and recombination (SPR1: co-pyrolysis of PDMS and PVC) of the mixture, resulting in the formation of pale-yellow PCS and PCS-C (**Figure S3b**). The SPR1-treated mixture was placed in an alkaline environment and inert gas environment to ensure that PCS and PCS-C could be oriented towards SiC generation during SPR2 (**Figure S3c**). During SPR2, PCS and PCS-C were heated to over 1000°C by a focused continuous-wave laser while an external electric field was applied, resulting in the preparation of SiC nanocrystals with abundant orientations (correspond to axial directions and dipole direction), types, and heights (**Figure S3d**). The crystal orientations and crystal types of SiC in different regions were determined by micro-area XRD with Laue method. By mapping the regions where the required crystal directions are located and preparing the corresponding spin structures, the encoding of the determined axial directions and dipole directions is realized. By mapping the regions where the required crystal types are located and preparing the corresponding spin structures, the encoding of the determined spin types is realized. We used the direct writing method of the focused femtosecond laser to realize the preparation of spin structure of color center (**Figure S3e**, **S4f**). By adjusting the heights of focal plane and laser pulse energy, the preparation of spin structures with different heights was realized, and the encoding of heights can be realized. After all the spin structures were prepared, the properties of spin structures at each position were mapped by ODMR, PL, Rabi oscillation and polarization spectra, and the unnecessary spin structures were removed by FIB and other methods, and the desired spin structure was finally obtained. Finally, a PDMS protective layer was applied to the SMED surface to protect the whole SMED structure (**Figure S3f**).

The parts required to be recombined are Si in PDMS and C in PVC (**Figure S4a**). During SPR1, a fly-eye lens is used to shape the Gaussian beam into a uniform beam. At ~450°C, PVC produces free (unsaturated) carbon through elimination reaction, and PDMS long chain is cleaved into PDMS short chain (**Figure S4b**). Subsequently, the short chain of PDMS reacts with free carbon, and carbon atoms are incorporated into the frame of PDMS through addition reaction. As a result, PCS and PCS-C generated by SPR1 are excellent precursors for SiC and SiC-C, which are conducive to the preparation of SiC with a high yield (**Figure S4c**). During SPR2, the inert gas environment avoids oxidation of the formed SiC above 1000°C, and the alkaline environment avoids excessive consumption of free carbon (**Figure S3d**). The external electric field makes the anions and cations move towards each other, increasing the contact between free carbon and silicon ions, and increasing the rate of SiC formation (**Figure S3e**).

In order to verify the seven-dimensional multiplexing of SMED, we prepared an array of 4×4 encoding units (SMED_4×4_), as shown in **Figure S3g, S3h**. The photoluminescence images of SMED_4×4_ was collected by a CCD camera, while the spin structures of SiC divacancy were excited by a 915nm laser (**Figure S3f**, **3i**).

The prepared samples were characterized by Raman spectroscopy, XRD, TEM and SAED, and the crystal properties in each microregion were mapped. Based on the optical micrographs and Raman spectra, we determined that the prepared SMED samples mainly consist of four regions: ①SiC region, ②SiOC and C mixture region, ③Denatured PDMS and escaping materials region, and ④PDMS region (**Figure S5a**). In terms of crystal orientation, type and height, the characterization results obtained by TEM and SAED are consistent with XRD, and it can be directly observed that there are SiC crystals with abundant orientations, types and heights (**Figure 2f, S5d-S5g**). We analyzed the spin structure properties of each microregion by ODMR spectra, Rabi oscillation curves, PL spectra and polarization spectra (**Figure S9-S12**). ODMR spectra proved that the crystal contained PL5, PL6 and other types of color center spin structures (**Figure S6a, S6b**). We characterized the Rabi oscillation effect of PL6 by using microwave pulse spin state regulation and measurement system, and the oscillation frequencies were ~1MHz and ~4MHz respectively (**Figure S6c**).


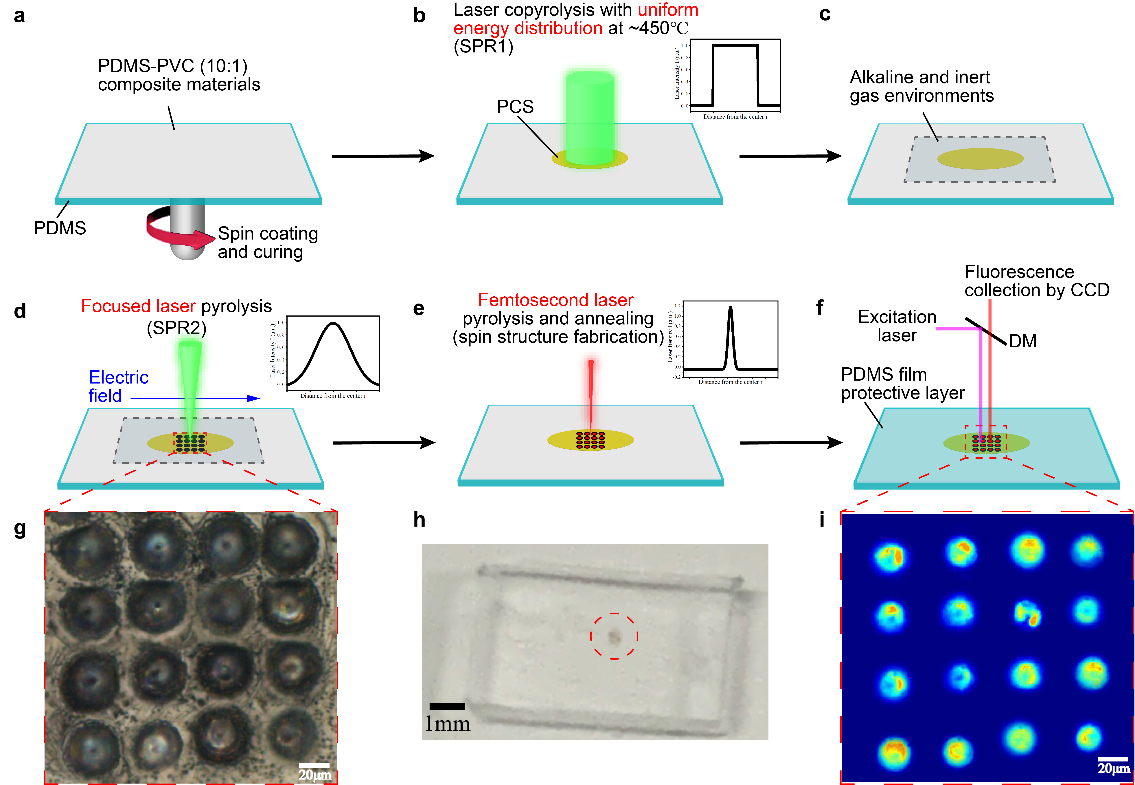


**Figure S3** Preparation and optical characterization of spin structures in SMED. **a,** Preparation of PDMS-PVC composites (precursors of PCS and PCS-C). **b,** SPR1: preparation of PCS and PCS-C (precursor of SiC). **c,** Environmental configuration with protective effect on SiC (inert gas and alkaline environment). **d,** SPR2: preparation of SiC. **e,** Preparation of SiC color centers by femtosecond laser. **f,** Preparation of protective layer and readout scheme of SiC color centers. **g,** Microscopic image of SMED_4×4_. **h,** Macroscopic image of SMED_4×4_. **i,** Fluorescence image of SMED_4×4_.


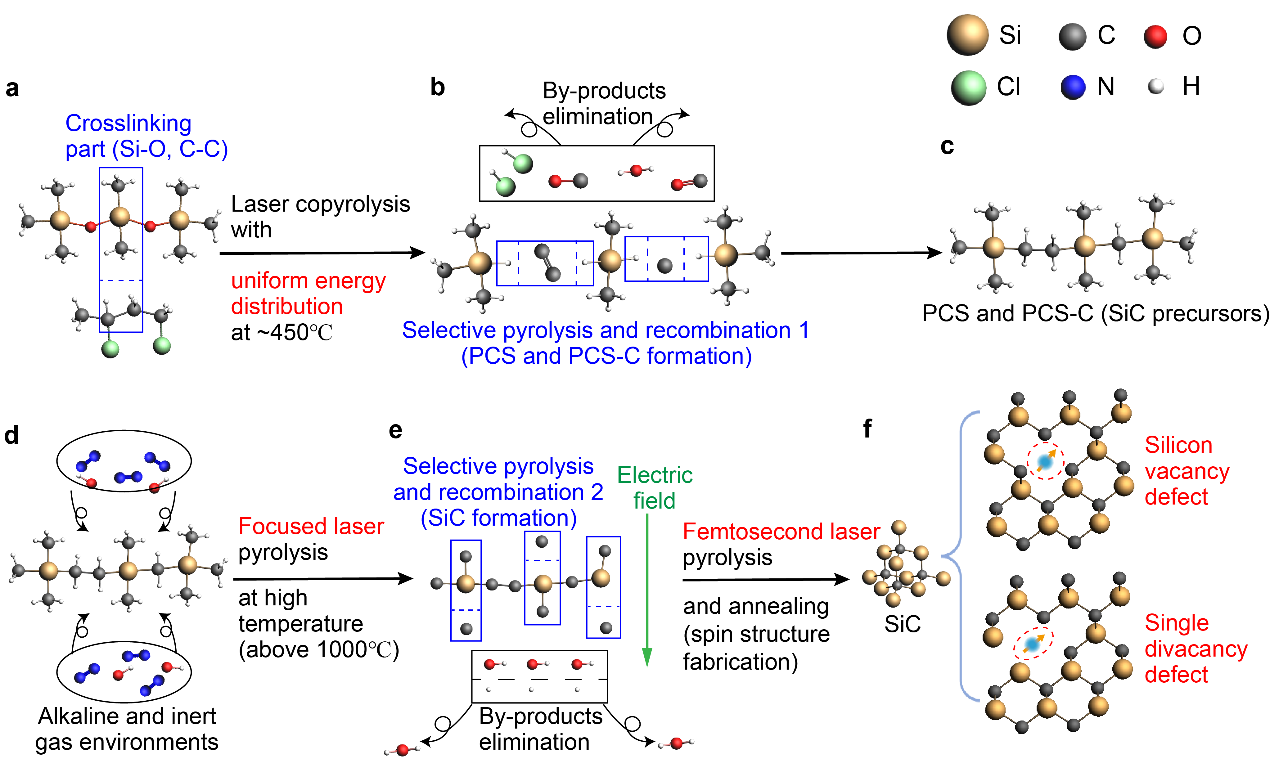


**Figure S4** Preparation of SiC and its spin structure at atomic and molecular level. **a,** PDMS-PVC mixture and its pyrolysis and recombination parts. **b,** SPR1: PDMS pyrolyzed into short chains, PVC underwent elimination reaction. **c,** PCS and PCS-C produced by SPR1. **d,** Configuration of inert gas environment and alkaline environment. **e,** SPR2: Pyrolysis of PCS and PCS-C and formation of SiC. **f,** Fabrication of SiC color centers using femtosecond laser.


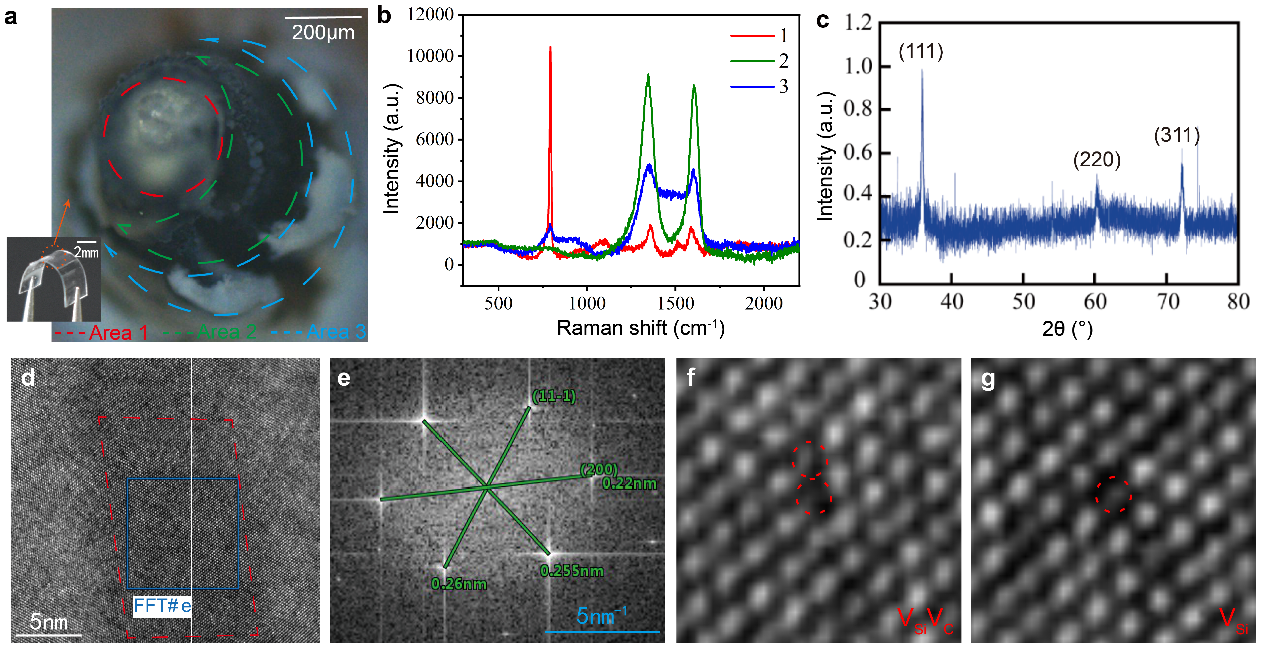


**Figure S5** Material characterization of PDMS-SiC composite. **a,** Macroscopic image and optical microscopic image of PDMS-SiC composites and their four material regions. **b,** Characterization of Raman Spectra in regions①-③. **c,** XRD spectrum of SiC crystals. **d-e,** TEM images (d) and corresponding SAED (e) images of SiC crystals. **f-g**, TEM images of silicon vacancy (V_Si_) and divacancy (V_Si_V_C_) of SiC nanocrystals prepared by laser direct writing.


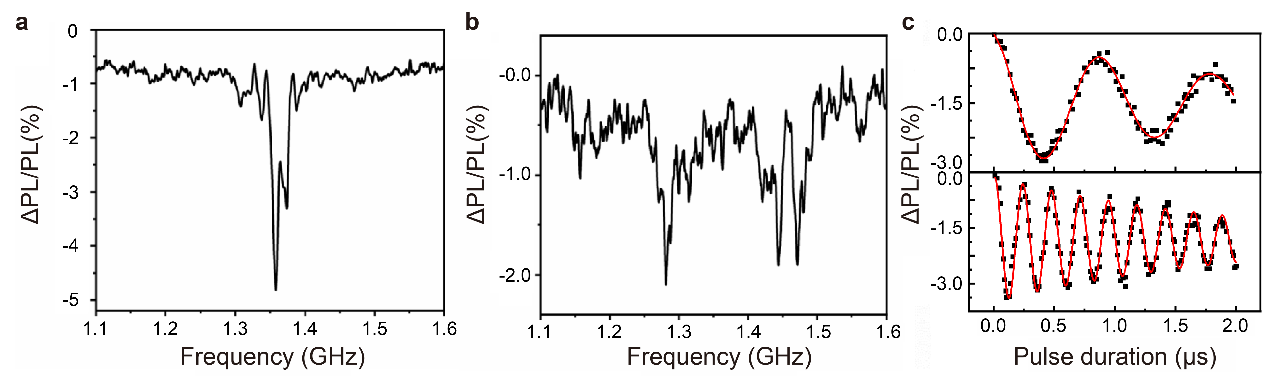


**Figure S6** ODMR spectrum (**a**, **b**) and Rabi oscillation curve (**c**) of SiC color centers in SMED.

**Note S5 Codes readout scheme and encoding performance verification**

A continuous encoded image (left part of **Figure S7**, usually 10-10^3^μm in size) can be decomposed into multiple independent encoding units (right part of **Figure S7**, usually less than 10μm in size). As shown in **Figure S8a**, **S8b**, we encoded a variety of images through SMED_4×4_ to demonstrate the image encoding ability of SMED (i.e., multiplexing ability on the X-axis and Y-axis). The internal structure of each encoding unit and its corresponding TEM results are shown in **Figure S8c** and **S8d** respectively. In the TEM image of the encoding unit, SiC crystals with various crystal orientations, crystal types and heights can be obviously observed, corresponding to the schematic diagram of crystal distribution. The ODMR spectra, Rabi oscillation spectra, PL spectra and polarization spectra in different regions (**Figure S6**, **S9-S12**) showed the rich color center spin structures in each encoding unit of SMED, as well as the rich axial directions, types, heights and dipole directions, corresponding to the schematic diagram of spin structure distribution.


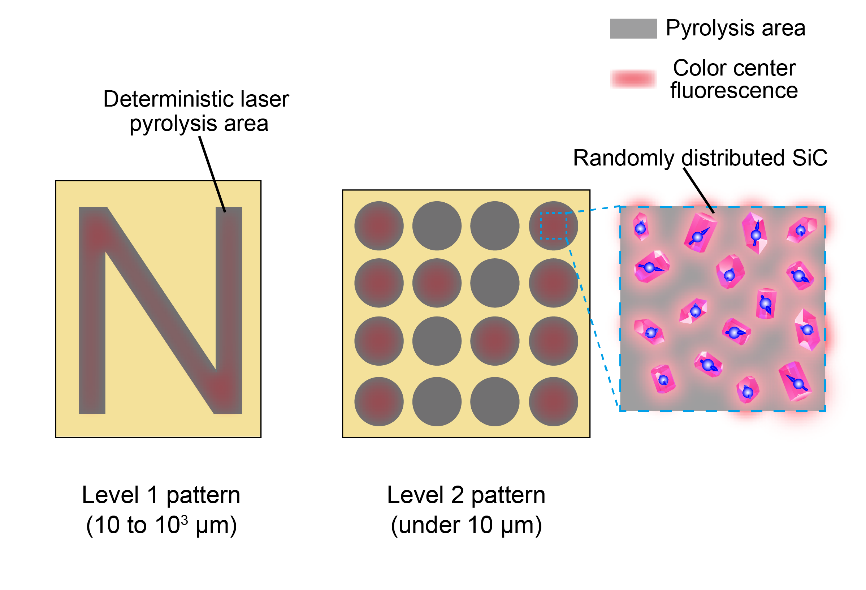


**Figure S7** Continuous encoded image composed of a large number of encoding units, discrete encoded image composed of a small number of encoding units, and SiC crystals in each encoding unit.


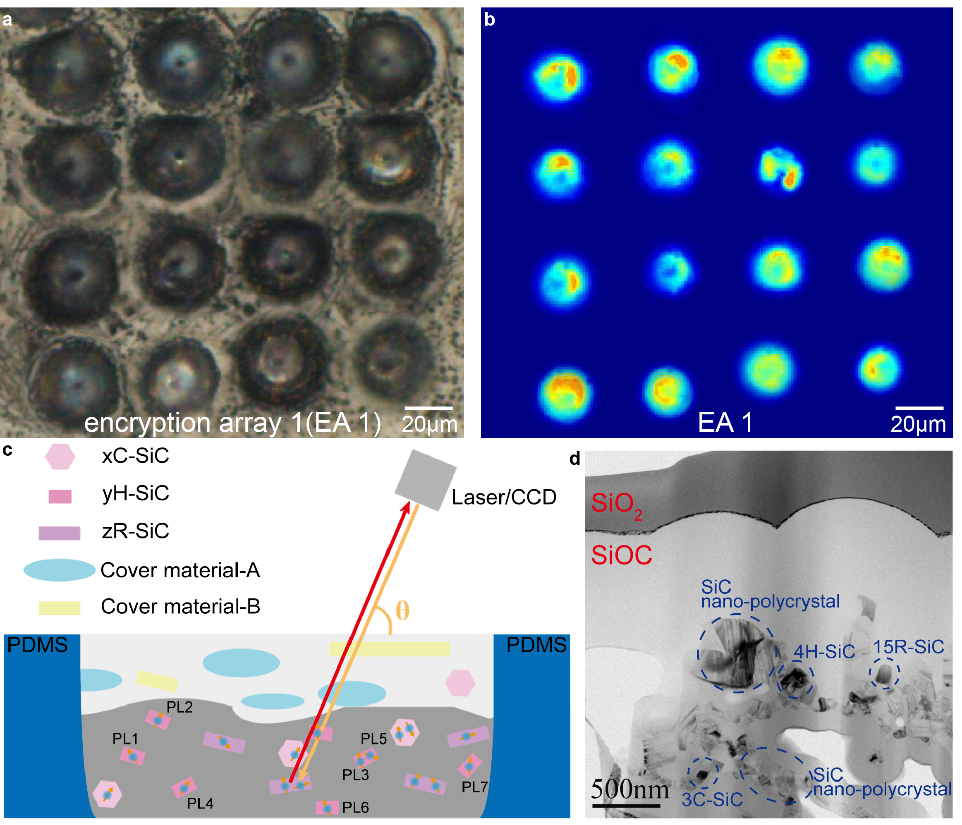


**Figure S8** Microscopic image (**a**), fluorescence image (**b**), encoding scheme (**d**) and decoding scheme (**c**) of SMED.

As the design scheme in the first part of the main text and **Note S2**, we analyzed each dimension while keeping the channels of the other three dimensions always the same and only changing the channels of the dimension that needed to be verified. In order to verify the code difference between channels of same encoding unit and the code difference between different encoding units, we selected two encoding units with obvious features in each dimension direction for verification.

When choosing the multiplexing channels of the magnetic field dimension, we mainly consider three criteria: 1. The distribution of resonance peaks (the existence of spin in a particular axial direction); 2. Linewidth of resonance peaks (to avoid crosstalk between magnetic field multiplexing channels); 3. Maximum achievable range of magnetic field strength (to ensure that all magnetic field multiplexing channels can be achieved). The axial directions of all spin structures in the sample are determined by curves in **Figure S9b**. According to **Figure S9b**, it can be seen that point 1 and point 2 have a variety of electron spins (the peaks) with different axial directions. Finally, considering the above criteria and the encoding requirements (three letters: NUC), we selected three magnetic field multiplexing channels (*M_1_*=18.5 Gs; *M_2_*=41.2 Gs; *M_3_*=64.5 Gs).

The ODMR spectra of magnetic field dimension and the code images of magnetic multiplexing channels are shown in **Figure S9b** and **S9c**, respectively. The microwave frequency is fixed at 1.45 GHz, and the filter central wavelength, microwave pulse duration, and polarization direction are set as *λ_1_*=1042.7 nm, *t_1_*=118 ns, *θ_1_*=0°, respectively. The red curve and the gray curve are the relationship curves between the PL intensity difference and the magnetic field intensity at point 1 (coordinates: (1, 1) as shown in **Figure S9c**) and point 2 (coordinates: (1, 2) as shown in **Figure S9c**), respectively. We took the baseline of each curve as its initial PL intensity (spin states are all |0>), and kept the values on the other three dimensions fixed. By selecting three magnetic field intensities (*M_1_*=18.5 Gs; *M_2_*=41.2 Gs; *M_3_*=64.5 Gs), three multiplexing channels (C_M, 1_= [*λ_1_*, *θ_1_*, *M_1_*, *t_1_*], C_M, 2_= [*λ_1_*, *θ_1_*, *M_2_*, *t_1_*], C_M, 3_= [*λ_1_*, *θ_1_*, *M_3_*, *t_1_*]) in magnetic field dimension were constructed, respectively. As shown in **Figure S9a**, point 1 exhibits obvious brightness changes when *M_1_* and *M_2_* are applied, but no brightness changes with *M_3_*; point 2 has obvious brightness changes when *M_3_* is applied, but no brightness changes with *M_1_* and *M_2_*. Thus, point 1 is encoded on C_M, 1_, C_M, 2_, C_M, 3_ channels as 1 (bright), 1 (bright), 0 (dark), respectively. And point 2 is encoded on C_M, 1_, C_M, 2_, C_M, 3_ channels as 0 (dark), 0 (dark), 1 (bright), respectively. Corresponding to the three magnetic field codes, the PL intensity difference diagram is shown at points 1 & 2 in **Figure S9c**. The brightness and coding of all points in the image are exactly consistent with the corresponding data results in **Figure S9b**. It proves that the magnetic dimension channel has good reuse ability.


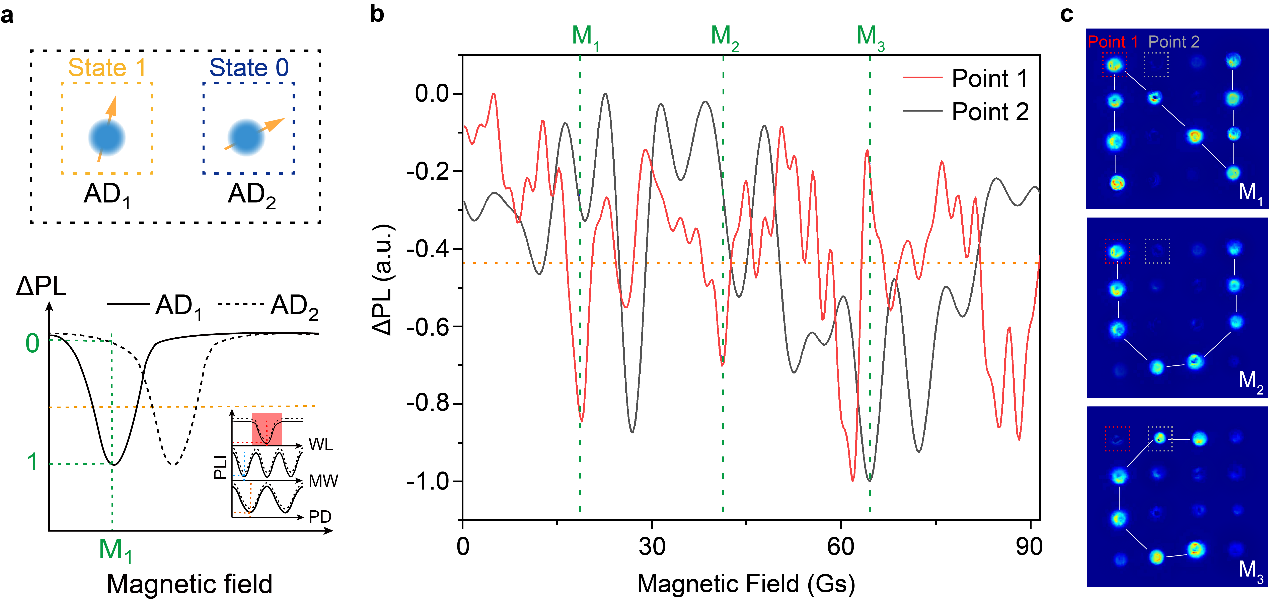


**Figure S9** The multiplexing capability of SMED in the magnetic field dimension. **a**, Spin properties and corresponding spectra used for encoding of magnetic field dimension. The spectra in main chart show different peak positions due to the different spin properties. The spectra in insert possess identical peak positions due to the same spin properties. **b**, magnetic field channel selection based on the fluorescence intensity difference. **c**, three coding maps corresponding to the three magnetic field multiplexed channels in (b).

The multiplexing coding results in wavelength dimension are shown in **Figure S10b** and **Figure** **S10c**, respectively. The microwave frequency was 1.45GHz, and the magnetic field intensity, microwave pulse time, and polarization direction were set as *M_1_*= 18.5Gs, *t_1_*= 118ns, and *θ_1_*=0°, respectively. As shown in **Figure S10b**, the gray curve and the red curve are the correlation curves between the fluorescence intensity difference and wavelength of point 1(coordinates: (1, 1) as shown in **Figure S10c**) and point 2(coordinates: (1, 2) as shown in **Figure S10c**), respectively, and the other three dimensions are fixed values. Two wavelengths (*λ_1_*= 1042.7 nm, *λ_2_*= 1131.5 nm) were selected to construct dimensions of two wavelength multiplexing channels (C_λ, 1_= [*λ_1_*, *θ_1_*, *M_1_*, *t_1_*], C_λ, 2_= [*λ_2_*, *θ_1_*, *M_1_*, *t_1_*]). As shown in **Figure S10a**, point 1 has an obvious brightness change at wavelength *λ_1_*, but no obvious brightness change at wavelength *λ_2_*. Point 2 has a significant brightness change at *λ_2_*, but no significant brightness change at *λ_1_*. Thus, point 1 is encoded on C_λ, 1_, C_λ, 2_ channels as 1 (bright), 0 (dark), respectively. And point 2 is encoded on C_λ, 1_, C_λ, 2_ channels as 0 (dark), 1 (bright), respectively. Corresponding to the two wavelength codes, the PL intensity difference diagram is shown at points 1 & 2 in **Figure S10c**. The brightness and coding of all points in the image are exactly consistent with the corresponding data results in **Figure S10b**. It proves that the wavelength dimension channel has good reuse ability.


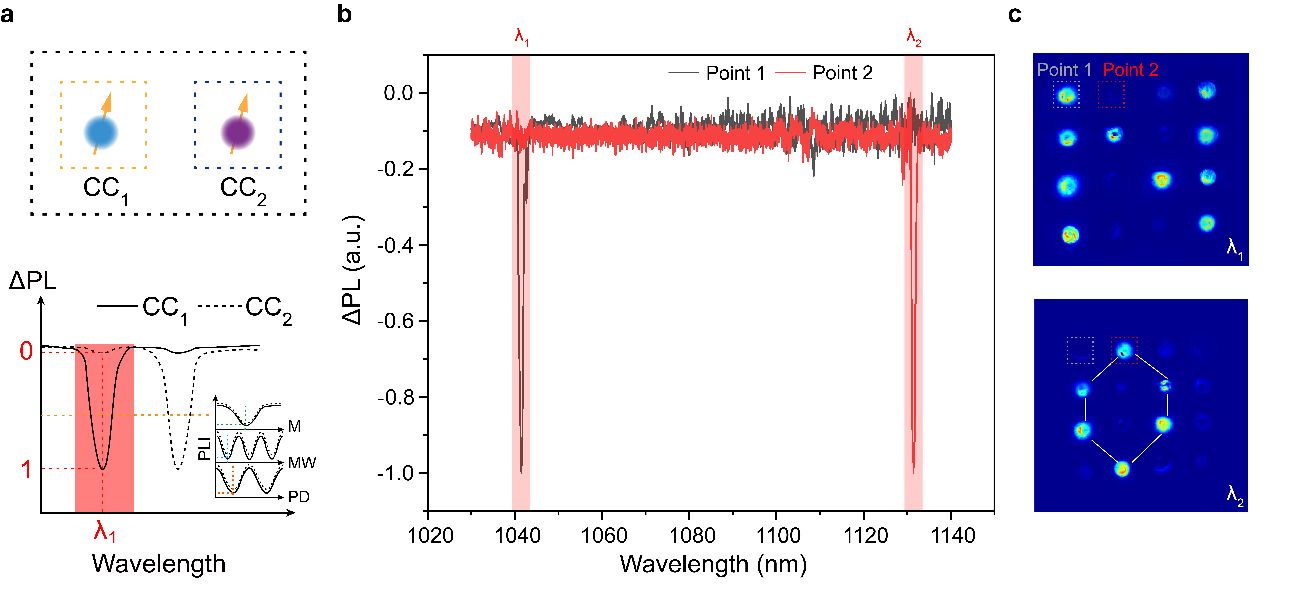


**Figure S10** The multiplexing capability of SMED in the wavelength dimension. **a**, Spin properties and corresponding spectra used for encoding of wavelength dimension. **b**, the correlation curve between fluorescence intensity difference and wavelength. **c**, two coding maps corresponding to the two wavelength multiplexed channels in (b).

The multiplexing coding results in ‘microwave pulse time’ (MPT) dimension is shown in **Figure S11b** and **S11d**, respectively. The microwave frequency was 1.45GHz, and the magnetic field intensity, wavelength, and polarization direction were set as *M_1_*=18.5 Gs, *λ_1_*=1042.7 nm, *θ_1_*=0°, respectively. As shown in **Figure S11b**, the top curve is the relationship curves between the PL intensity difference and the microwave pulse time at point 3 (coordinates: (1, 3) as shown in **Figure S11c**). The lower curve is the relationship curve between the PL intensity difference and the microwave pulse time at point 4 (coordinates: (1, 4) as shown in **Figure S11c**), and the other three dimensions are fixed values. Two microwave pulse times (*t_1_*=118 ns, *t_2_*=475 ns) were selected to construct dimensions of two MPT multiplexing channels (C_t, 1_= [*λ_1_*, *θ_1_*, *M_1_*, *t_1_*], C_t, 2_= [*λ_1_*, *θ_1_*, *M_1_*, *t_2_*]). As shown in **Figure S11b**, point 3 has an obvious brightness change at microwave pulse time *t_2_*, but no obvious brightness change at *t_1_*. Point 4 has a significant brightness change at *t_1_*, but no significant brightness change at *t_2_*. Thus, point 3 is encoded on C_t, 1_, C_t, 2_ channels as 0 (dark), 1 (bright), respectively. And point 4 is encoded on C_t, 1_, C_t, 2_ channels as 1 (bright), 0 (dark), respectively. Corresponding to the two MPT codes, the PL intensity difference diagram is shown at points 3 & 4 in **Figure S11c**. The brightness and coding of all points in the image are exactly consistent with the corresponding data results in **Figure S11b**. It proves that the MPT dimension channel has good reuse ability.


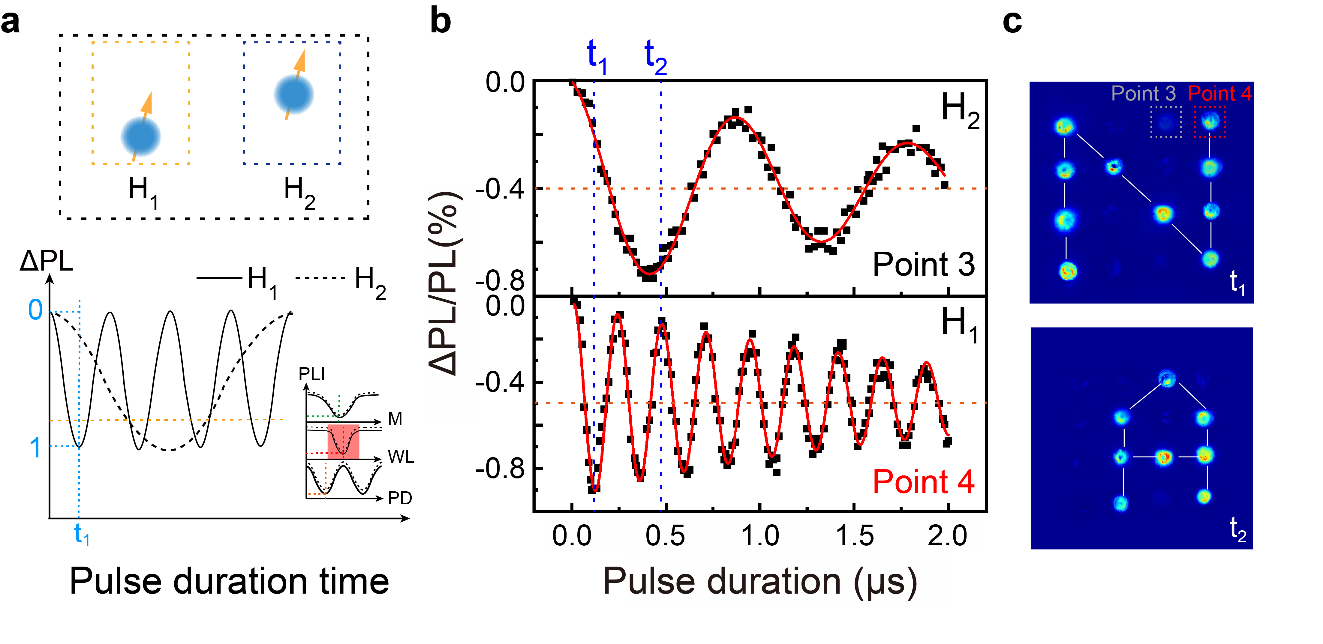


**Figure S11** The multiplexing capability of SMED in the MPT dimension. **a**, Spin properties and corresponding spectra used for encoding of MPT dimension. **b**, the correlation curve between fluorescence intensity difference and microwave pulse time. **c**, two coding maps corresponding to the two MPT multiplexed channels in (b).

The multiplexing coding results in polarization dimension are shown in **Figure S12b** and **Figure** **S12c**, respectively. The microwave frequency was 1.45GHz, and the magnetic field intensity, filter wavelength, and microwave pulse time were set as *M_1_*=18.5 Gs, *λ_1_*=1042.7 nm, *t_1_*=118 ns, respectively. As shown in **Figure S12b**, the top curve is the relationship curve between the PL intensity difference and the polarization angle at point 7 (coordinates: (2, 3) as shown in **Figure S12c**). The lower curve is the relationship curve between the PL intensity difference and the polarization angle at point 11 (coordinates: (3, 3) as shown in **Figure S12c**), and the other three dimensions are fixed values. Two polarization angles (*θ_1_*=0°, *θ_2_*=90°) were selected to construct dimensions of two polarization multiplexing channels (C_θ, 1_= [*λ_1_*, *θ_1_*, *M_1_*, *t_1_*], C_θ, 2_= [*λ_1_*, *θ_2_*, *M_1_*, *t_1_*]). As shown in **Figure S12a**, point 7 has an obvious brightness change at polarization angle *θ_2_*, but no obvious brightness change at *t_1_*. Point 4 has a significant brightness change at *t_1_*, but no significant brightness change at *θ_1_*. Thus, point 7 is encoded on C_θ, 1_, C_θ, 2_ channels as 0 (dark), 1 (bright), respectively. And point 11 is encoded on C_θ, 1_, C_θ, 2_ channels as 1 (bright), 0 (dark), respectively. Corresponding to the two polarization angles codes, the PL intensity difference diagram is shown at points 7 & 11 in **Figure S12c**. The brightness and coding of all points in the image are exactly consistent with the corresponding data results in **Figure S12b**. It proves that the polarization dimension channel has good reuse ability.


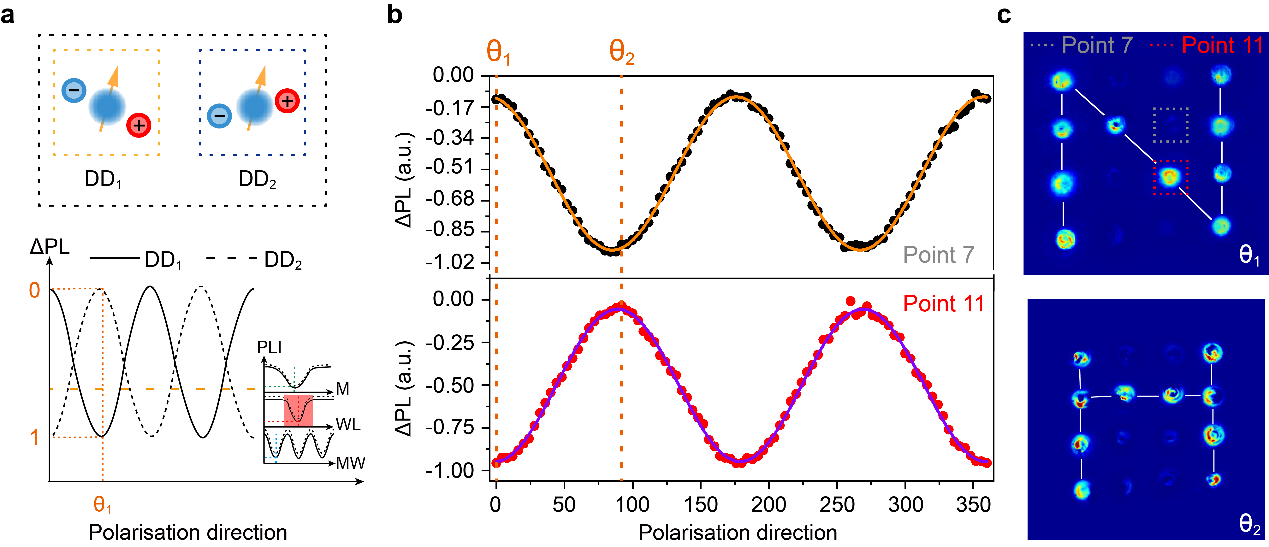


**Figure S12** The multiplexing capability of SMED in the polarization dimension. **a**, Spin properties and corresponding spectra used for encoding of polarization dimension. **b**, the correlation curve between fluorescence intensity difference and polarization angles. **c**, two coding maps corresponding to the two polarization multiplexed channels in (b).

In theory, the five additional dimensions and their multiplexing channels are independent of each other (as described in the section **Mechanism of Spin-multiplexing** of maintext). However, in the experiment and application process, due to the non-uniformity of each device, the cross-talk between the devices, and the existence of the redundant spin structures, the independence of each dimension is weakened (there is a certain correlation). In terms of experimental results, the above correlation is mainly reflected in the fact that some points coded as 0 still have certain PL changes (**Figure S9-S12**). Therefore, further optimization of experimental equipment and removal of the existence of redundant spin structures will help to avoid the reduction of information storage density caused by correlation.

**Note S6 Coding information processing**

After collecting the fluorescence intensity difference signal, we segment and binarize the fluorescence intensity difference signal to extract the final coding information. According to the position of the encoded pixel, the fluorescence difference image is divided into 16 regions, each region is a pixel unit containing multiple sub-pixels. In binarization, 0.4 times of the maximum fluorescence intensity difference is set as the threshold for binarization. The binarized image corresponding to the coded image in **Figure 3e** is shown in **Figure S13**, where black represents the coded 1 and white represents the coded 0.


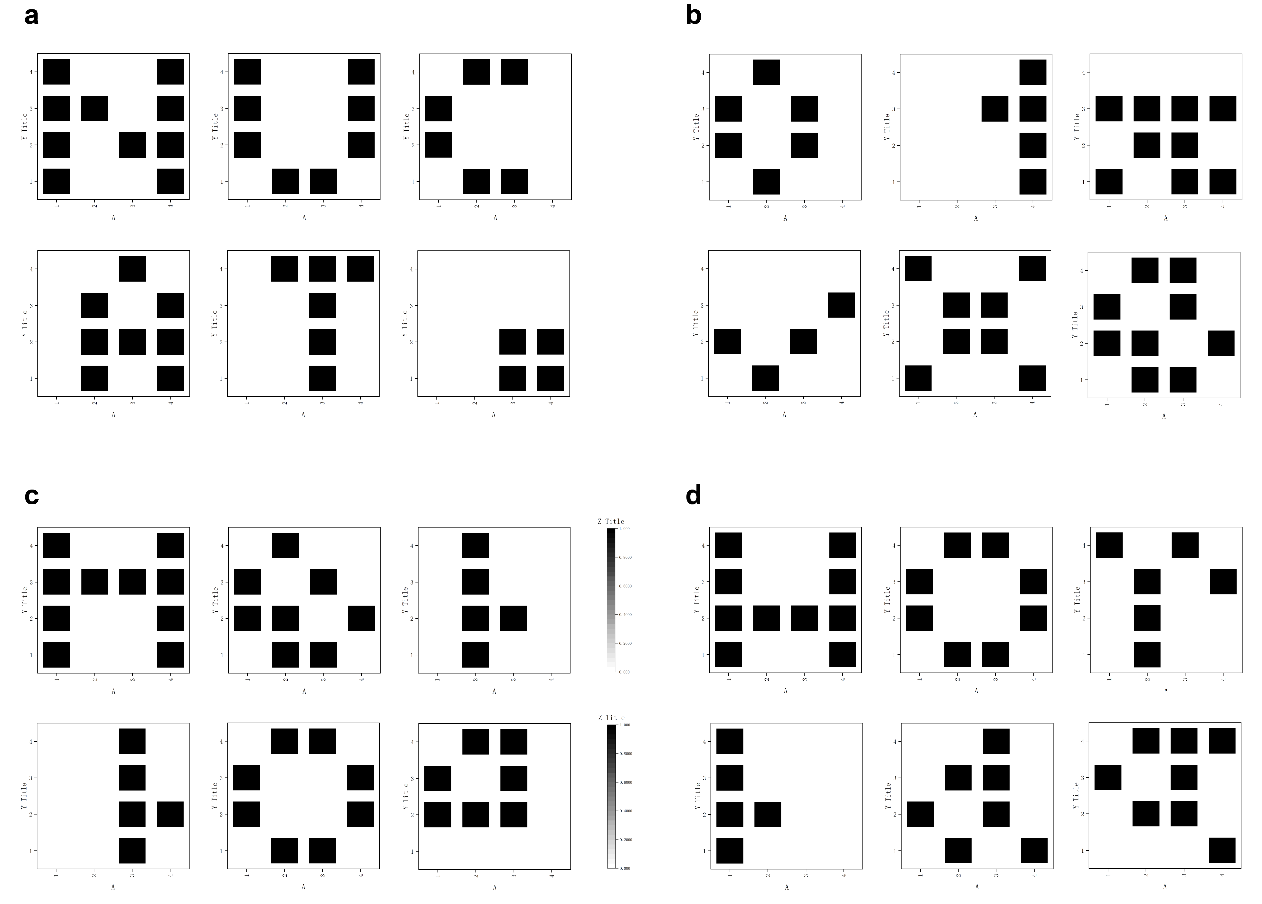


**Figure S13** The segmented and binarized encoded image information (corresponding to the fluorescent image in **Figure 3e**).

**Note S7 SMED device**

SMED equipment is integrated by four kinds of functional structures, including PDMS film embedded with SIC nanocrystals, microwave waveguide structure, planar magnetic coil, and micro-optical filter structure. In addition to the spin structure layer, the other three layers are the three-dimensional multiplexed channel coding control layer of the spin structure. The polarization control layer, which is integrated into the signal excitation or collection end, and finally SMED device has been integrated. The four coding control layers are electrically controlled.

As shown in **Figure S14,** the microwave waveguide layer, the planar magnetic coil layer and the micro-optical filter layer are all manufactured by micro-nano machining technology. The microwave waveguide structure is a toroidal metal waveguide antenna, which is depositing metal on the PI substrate with a thickness of 200nm and photoengraved into a toroidal waveguide antenna (linewidth of 150μm, inner diameter of 420μm). Then, it is connected to an external microwave source by lead bonding through the lead-out electrode. The planar magnetic coil layer is manufactured by depositing Permalloy film on PI substrate, and then by lithography and stripping process. The planar magnetic coil structure is composed of a Permalloy ring conductor (line width of 300μm, inner diameter of 350μm, thickness of 100nm) and a Permalloy core (radius of 130μm) in the coil. Due to the extremely high magnetic susceptibility (~10^5^) and the extremely high saturation magnetic induction intensity (0.6~1.0T), Permalloy can provide an adequate magnetic field for SMED at a small magnetizing current. The micro-optical filter layer is composed of a liquid crystal tunable filter, which has a narrow bandwidth (<10 nm) and fast switching speed. It enables high-speed electronically controlled SMED and effective filtration of SMED fluorescence.


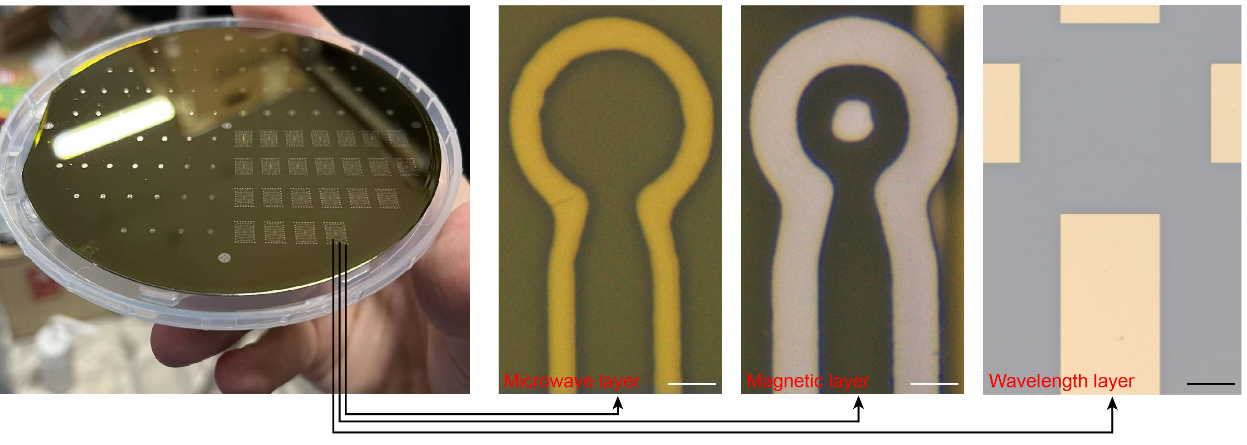


**Figure S14** SMED (integrated microwave waveguide structure, planar magnetic coil, and micro-optical filter structure) (scale bar: 300μm).

The polarization angle of spin fluorescence is controlled by using the liquid crystal polarization direction rotator (Liquid Crystal Polarization Rotators, custom made at Lbtek) integrated in the collecting end and the polarizer at the back end. When the pump light is circularly polarized, the color center structure of all dipole directions is excited together, and the emitted fluorescence has multiple polarization angles. By controlling the voltage of LCPR, the polarization direction of spin fluorescence is rotated perpendicular or parallel to the polarization direction of the polarizer. In this way, the fluorescence signal of a certain polarization direction is selected to output, and the polarization direction of the fluorescence signal is encoded by reading out. Due to the limitation of the polarization direction of the input light by the LCPR device, it mainly realizes the coded readout of the four polarization directions of 0°, 90°, 180° and 270°.

In addition, when the color center is pumped by linearly polarized light, the pumped light is first converted to linearly polarized light with a polarizer. Control the polarization direction of the pump light by LCPR, the polarization direction of the linearly polarized pump light can be continuously rotated more than 180°. Because different kinds of color centers can only be excited by the pump light of a specific polarization direction, more polarization directions can be encoded by the above scheme as more kinds of color centers are manufactured.

**Note S8 Dynamic performance and encryption application**

The dynamic performance of the control layer structure and the control device in SMED devices are tested, respectively. The relationship curve between the bit error rate and the control frequency of magnetic field, wavelength, and microwave equipment is shown in **Figure S15b**. The control frequency of magnetic field equipment is mainly limited by current switching time and magnetization time. The control frequency of the wavelength device is mainly limited by the voltage switching time and the liquid crystal state switching time. Because the above four kinds of time can reach the microsecond or nanosecond level, magnetic field equipment and wavelength equipment have high-dynamic frequencies. With a tolerance of bit error rates of 0.5% and 1.5%, the dynamic frequencies are 1.4 kHz and 570.8 kHz, respectively. The control frequency of microwave equipment is mainly limited by switching time, microwave transmission time, and microwave pulse train time. Because the circuit is short and the thickness of the SMED device is thin, the switching time and microwave transmission time can reach the nanometer level. However, due to the longtime of microwave pulse sequences and the multiple sequence combinations for quantum state regulation, the dynamic performance of microwave devices is slightly lower than that of magnetic field and wavelength devices. With a tolerance of bit error rates of 0.9% and 2.0%, the dynamic frequencies are 1.4 kHz and 570.8 kHz, respectively.

In the encryption application shown in **Figure 4b** and **S15c**, Real-time encryption of optical images was achieved by the magnetic field (M) dimension, wavelength (λ) dimension, and microwave duration time (t) dimension with fast response speed (multiplexing channel switching speed). The fast response speed makes the above three dimensions have advantages for application scenarios with fast encryption requirements. In addition, we validate the results of polarization (θ) dimension and Z dimension for fast encryption applications. As shown in **Figure S15d**, because the multiplexing channel switching speed of θ dimension and Z dimension is slower than the image acquisition speed, some image frames cannot be encrypted. Therefore, the above negative results prove that the θ dimension and the Z dimension have no advantages for fast image encryption application described in **Figure 4a**.


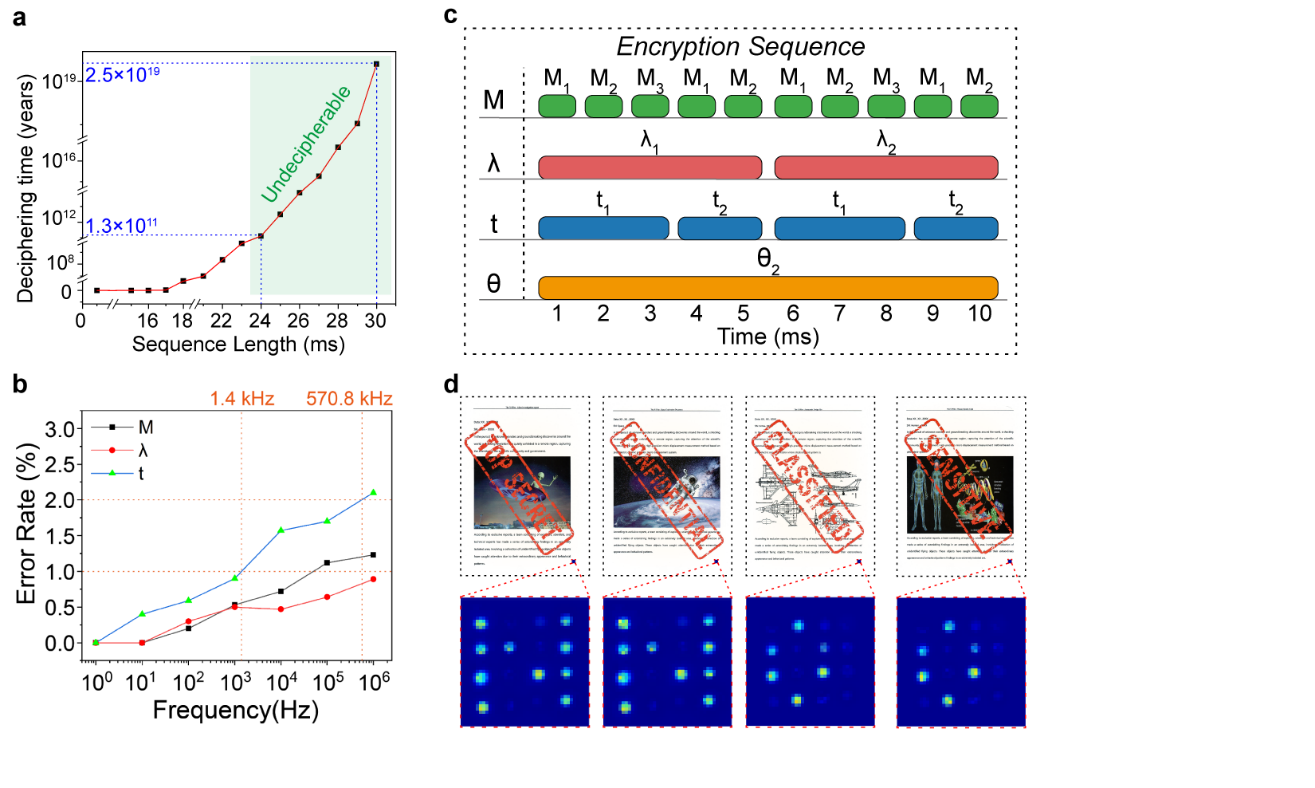


**Figure S15 a,** The deciphering time corresponding to a certain encryption sequence length. **b**, The dynamic performance of the waveguide layer of microwave excited structure, the planar magnetic coil layer and the micro-optical filter layer of SMED device. **c**, Encryption sequence for system verification. **d**, Imaging results of secret document encryption based on polarization dimension and Z dimension.

Movie S1.

The encryption/watermarking process of electronic documents.

Movie S2.

The encryption/watermarking process of paper documents.
